# Quantifying Dynamic Phenotypic Heterogeneity in Resistant *Escherichia coli* under Translation‐Inhibiting Antibiotics

**DOI:** 10.1002/advs.202304548

**Published:** 2024-01-09

**Authors:** Haishuang Zhu, Yixiao Xiong, Zhenlong Jiang, Qiong Liu, Jin Wang

**Affiliations:** ^1^ State Key Laboratory of Electroanalytical Chemistry Changchun Institute of Applied Chemistry Chinese Academy of Sciences Changchun Jilin 130022 China; ^2^ School of Applied Chemistry and Engineering University of Science and Technology of China Hefei Anhui 230026 China; ^3^ Department of Chemistry Physics and Applied Mathematics State University of New York at Stony Brook. Stony Brook New York 11794‐3400 USA

**Keywords:** bacterial antibiotic resistance, gene expression network, growth rate, landscape and flux theory, phenotypic heterogeneity, quantitative dynamics

## Abstract

Understanding the phenotypic heterogeneity of antibiotic‐resistant bacteria following treatment and the transitions between different phenotypes is crucial for developing effective infection control strategies. The study expands upon previous work by explicating chloramphenicol‐induced phenotypic heterogeneities in growth rate, gene expression, and morphology of resistant *Escherichia coli* using time‐lapse microscopy. Correlating the bacterial growth rate and cspC expression, four interchangeable phenotypic subpopulations across varying antibiotic concentrations are identified, surpassing the previously described growth rate bistability. Notably, bacterial cells exhibiting either fast or slow growth rates can concurrently harbor subpopulations characterized by high and low gene expression levels, respectively. To elucidate the mechanisms behind this enhanced heterogeneity, a concise gene expression network model is proposed and the biological significance of the four phenotypes is further explored. Additionally, by employing Hidden Markov Model fitting and integrating the non‐equilibrium landscape and flux theory, the real‐time data encompassing diverse bacterial traits are analyzed. This approach reveals dynamic changes and switching kinetics in different cell fates, facilitating the quantification of observable behaviors and the non‐equilibrium dynamics and thermodynamics at play. The results highlight the multi‐dimensional heterogeneous behaviors of antibiotic‐resistant bacteria under antibiotic stress, providing new insights into the compromised antibiotic efficacy, microbial response, and associated evolution processes.

## Graphical abstract

This study explores dynamic phenotypic heterogeneity in antibiotic‐resistant bacteria under chloramphenicol treatment. By dissecting and integrating the heterogeneity in growth rates, gene expressions, and morphologies, novel cell fates are uncovered, and the underlying mechanisms are elucidated using a gene expression network model. Moreover, the quantitative analysis provides valuable insights into the dynamics and thermodynamics of the antibiotic‐resistant bacteria under chloramphenicol treatment.

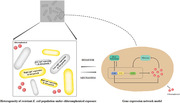

## 1 Introduction

Phenotypic heterogeneity^[^ ^1^, ^2^, ^3^ ^]^ is a phenomenon in which diverse phenotypes emerge in isogenic populations under homogeneous conditions, indicating a “bet‐hedging” strategy that promotes adaption or evolution in response to unpredictable environmental changes.^[^ ^4^, ^5^ ^]^ The behaviors of “labor division” are manifested in growth,^[^ ^6^, ^7^ ^]^ gene expression,^[^ ^8^, ^9^, ^10^ ^]^ metabolism,^[^ ^11^, ^12^ ^]^ and the other heterogeneities,^[^ ^13^, ^14^, ^15^ ^]^ among which an internal connection may exist.^[^ ^16^ ^]^ Fluctuations or noises are considered the original driving force behind phenotypic heterogeneity, while the presence of regulatory motifs can amplify or suppress its effects.^[^ ^17^ ^]^ Multiple cell fates can be encoded by observing more than one product with internal regulations.^[^ ^18^ ^]^ For example, a recent study has revealed that four production states for two mutually repressing gene circuits represent a classic bistable genetic switch of lambda phage,^[^ ^19^ ^]^ expanding cell fate potentials beyond the classical picture of bistable switches. Furthermore, quantifying the transitions among phenotypic states allows for elucidating the dynamic switching pathways and the underlying intra‐molecular mechanisms.

The emergence of antibiotic resistance is a common and concerning issue when treating bacterial infections. Under antibiotic stress, bacterial populations may develop resistance and exhibit phenotypic heterogeneities, enabling a fraction of the cells to persist in harsh environments and consequently leading to recurrent infections. In such conditions, growth rate bistability may emerge, where a dormant state and a growing state co‐exist. The dormant state cells are conventionally viewed as more tolerant to antibiotics.^[^ ^16^, ^20^ ^]^ They can restore the bacteria population under favorable conditions, utilizing a “bet‐hedging” strategy to avoid arrestment by antibiotics and perpetuate the infection. The global regulation of the gene expression levels in bacteria is closely tied to their growth rate. This relationship arises due to the impact of the cellular parameters, such as the abundance of RNA polymerases and ribosomes, which are influenced by the growth rate and, in turn, exert influences on gene expressions.^[^ ^21^ ^]^ The relationship between bacterial gene expressions and growth rate encompasses a multitude of possibilities that are contingent upon the environmental context and regulatory mechanisms at play. Under nutrient‐limited conditions, a gene expression bistability with low and high expression states can be observed within a typical positive feedback gene regulation motif due to the influence of growth rate. A high growth rate leads to a low expression state because of the dominant dilution effect, while a low growth rate leads to a high expression state because of its accumulative effect.^[^ ^22^ ^]^ Under translational inhibition conditions, like chloramphenicol (Cm)^[^ ^21^ ^]^ and macrolide,^[^ ^23^ ^]^ however, opposite results may occur owing to the affected ribosome activities.

Cm is a broad‐spectrum antibiotic that acts by inhibiting mRNA translation on the 50S ribosome during the peptide bond formation process.^[^ ^24^ ^]^ This renders Cm capable of exerting a global translation suppression effect on both regulatory and constitutive genes by combining with and inactivating the ribosome. Meanwhile, the presence of chloramphenicol acetyltransferase (CAT) can detoxify Cm by catalyzing the acetyl‐S‐CoA‐dependent acetylation of Cm^[^ ^25^ ^]^ and preventing it from binding to ribosomes.^[^ ^26^ ^]^ In the event of a bacterial strain developing a certain degree of Cm resistance, the reduced inhibitory effect of Cm would result in a higher level of expression of the global genes. This heightened gene expression, exemplified by CAT, leads to a higher degree of resistance (repressing more Cm) and thereby encourages the expression of the gene products in a positive feedback loop. We aim to investigate the presence of the gene expression heterogeneity and its potential correlation with the growth rate heterogeneity or multi‐modality within the context of this Cm‐CAT positive feedback loop. To address this objective, we have selected cspC as our target gene and conducted the experiments at 37 °C to eliminate temperature‘s influence on this cold shock gene. The choice of the gene cspC stems from its characteristic constitutively high expression levels, which enable us to effectively discern the fluorescence of its reporter gene under the global expression suppression effect of high concentrations of Cm. Additionally, cspC is recognized as one of the stress response genes,^[^ ^27^ ^]^ known to be strongly induced by antibiotics^[^ ^28^ ^]^ and capable of up‐regulating RNA polymerase activity,^[^ ^29^ ^]^ thereby also likely enhancing the reporter gene expression. Given these considerations, we pose several key inquiries: Does the expression of cspC manifest heterogeneity, and does the previously reported correlation between the bacterial growth rate and gene expression endure within this specific context? Moreover, what underlying mechanisms govern these complex behaviors?

In this study, we endeavored to discern multi‐dimensional phenotype heterogeneities within resistant *Escherichia coli (E. coli)* populations that had been subjected to various concentrations of Cm treatment. We accomplished this by employing a combination of analytical methods based on steady‐state and real‐time microscopic observations, including quantifying cspC gene expressions via the translationally infused YFP fluorescence intensity,^[^ ^30^ ^]^ evaluating cell morphologies by area, and assessing cell physiology through the growth rate, as determined by the cell cycle. Our methodology facilitated the identification of heterogeneity within each dimension and allowed us to investigate the multidimensional heterogeneity by combination of different traits. By correlating the cspC gene expression with the growth rate, we observed a counterintuitive four‐state heterogeneity in the sub‐MIC antibiotic concentrations. To explain the underlying mechanisms driving this behavior, we put forth a concise gene expression network model. To gain further insights into the dynamic behaviors of the phenotypes across diverse Cm concentrations, we applied a modified Hidden Markov Model (HMM), often used for temporal pattern recognition,^[^ ^31^ ^]^ to fit the real‐time single‐cell data trajectories of gene expression fluorescence intensity,^[^ ^19^, ^32^, ^33^ ^]^ morphology,^[^ ^34^ ^]^ and growth rate.^[^ ^35^ ^]^ Based on the HMM fitted results, we examined observable behaviors such as residence time and switching time, as well as the non‐equilibrium dynamics and thermodynamics involved.^[^ ^36^ ^]^ Specifically, our analysis of non‐equilibrium dynamics and thermodynamics included the potential landscape topography, state switching paths and barrier heights, flux loop values, time irreversibility, and entropy production rate. The consistency among our experimental and calculated results not only manifested the role the non‐equilibrium played on dynamical instability, time irreversibility, and thermodynamic cost but also revealed a relationship between available physical quantities and cellular physiology.

## 2 Results

### 2.1 Two Gene Expression Subpopulations Emerged upon Sub‐MIC Cm Treatment

**Figure 1:**
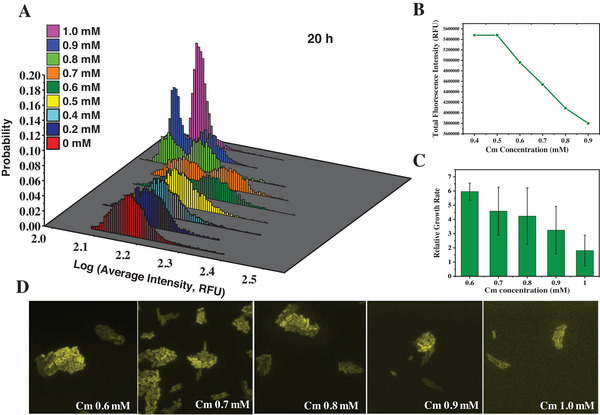
Observation of expression bistability at 20 h following various concentrations of Cm treatment cultured in batch. A) With increasing concentrations of Cm from 0.4 to 1.0 mm in the FCS2 chamber, the resulting YFP expression intensities and distributions at 20 h were dependent on the concentrations of Cm. Six colors of histograms represent different Cm concentrations. B) The total fluorescence intensity changes indicated by the product of average intensities and its area at concentrations vary from 0.4 to 0.9 mm. 2000 cells were collected and analyzed for each concentration. C) The average relative growth rate under increasing concentrations of Cm. The error bar for each concentration indicated the dispersion degree of growth rate. The results were obtained from growth analysis of eight microcolonies over a 30‐h period. D) Representative fluorescence image of the cells captured under different concentrations of Cm at 20 h with a time‐lapse microscope.

We employed a YFP‐reporting system within the cspC gene in the *E. coli* K‐12 model strain with Cm resistance to investigate the heterogeneity of expression. We collected both in batch (after 10 or more hours of antibiotic treatment) and real‐time data using a fluorescence microscope following treatment with varying concentrations of Cm. The microscopic observation of bacterial cells was conducted using the Focht Chamber System 2 (FCS2, Bioptechs) chamber. As depicted in **Figure** **1**A, we analyzed the gene expression distributions at the 20th h after treatment with concentrations of Cm ranging from 0 to 1.0 mm. After performing quantitative image analysis (see Experimental Section),^[^ ^33^ ^]^ we identified two expression sub‐populations that were clearly distinguishable at sub‐minimum inhibitory concentration (sub‐MIC) levels of Cm (i.e., ranging from 0.6 to 0.9 mm, MIC value = 1.0 mm).^[^ ^6^ ^]^ In contrast, no such bimodal behavior was observed in the control groups with Cm concentrations <0.6 mm at multiple time points. (see Figure 1A; Figure S1A, Supporting Information). These observations indicated that the occurrence of the bistability in cspC expression was not a random phenomenon but rather dependent on the specific conditions. Therefore, we focused on the heterogeneous behavior of our model strain following 0.6, 0.7, 0.8, and 0.9 mm of Cm treatment in particular. As the concentration of Cm increased, the total fluorescence intensity of the cells gradually decreased (Figure 1B), which was due to the suppressed translational capacity of the ribosome.^[^ ^37^ ^]^ Furthermore, the average relative growth rate of the cells decreased as the concentration of Cm increased (Figure 1C). The error bars in **Figure** 1C represent the growth rate fluctuation extent or the coefficient of variation among the micro‐colonies, which could indicate the level of growth rate heterogeneity. These results revealed that the growth rate variation was highest under 0.8 mm Cm induction. Typical fluorescence images of the bacteria populations for each concentration are presented in Figure 1D, which illustrates significant fluorescence intensity differences at the single‐cell level. Notably, both the bright and dim portions of the cells were dimmer when cultured in higher concentrations of Cm.

**Figure 2:**
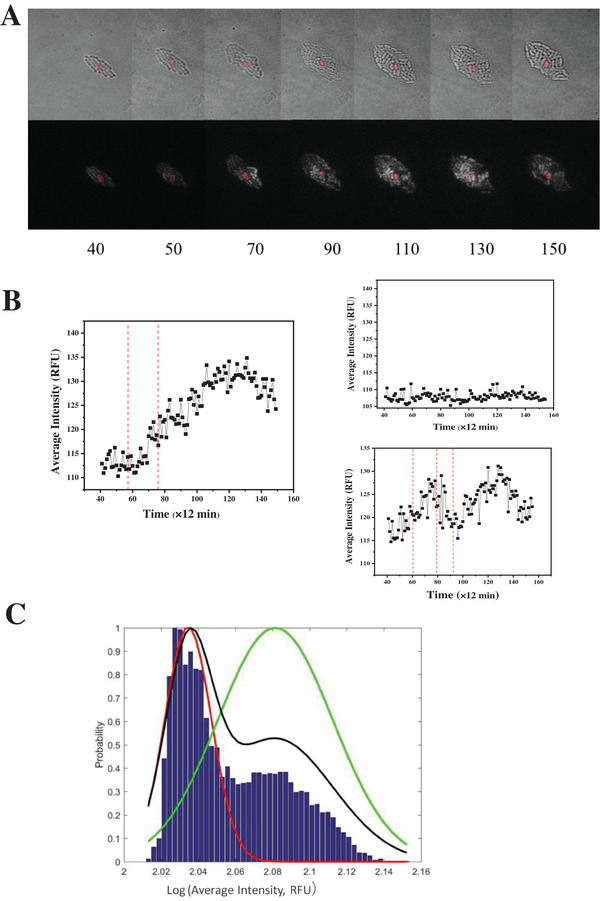
The real‐time single‐cell average fluorescence intensity trajectories following 0.9 mm Cm treatment. A) The bright field and fluorescence field images of a micro‐colony from a time‐lapse microscopy experiment. The time interval set for the microscopy image capture was 12 min. The number below the image represented the sequence of the time intervals. B) The YFP reporter fluorescence intensity trajectory shown in the left panel corresponded to the circled cell shown in (A); the two trajectories shown in the right panel were two other representative cells selected for demonstration. The vertical dashed line indicated the cell division events. C) The histogram showed the average intensity distribution of 6127 fluorescence points from 69 real‐time trajectories induced by 0.9 mm Cm. The three solid curves are the normalized output probability distributions acquired by CHMM fitting. The red and green lines refer to the normalized output probabilities of the low and the high expression states, respectively. The black line represents the total normalized output probability.

### 2.2 Real‐Time Monitoring of Fluorescence Intensity Revealed the Dynamical Changes of the Two Cell Fates

In order to gain deeper insights into the kinetic mechanism responsible for the observed two‐state behavior, we conducted real‐time observations of single colonies at the single‐cell level using the FCS2 microfluidic device in combination with fluorescence time‐lapse microscopy.^[^ ^38^ ^]^ We quantified the single‐cell cspC gene expressions by the average fluorescence intensity and analyzed the distribution of average fluorescence intensity values through large‐scale image statistics. The bacteria cells were monitored over time intervals of 12 min (0.2 h) for over 30 h. Figure 2A shows the representative images of a micro‐colony under 0.9 mm Cm treatment at prolonged periods of time. Both dim and bright cells could be seen in the same field and the intensity fluctuations were recorded. (Movies S1 and S2, Supporting Information). Three typical fluorescence intensity trajectories of cells under 0.9 mm Cm treatment are presented in Figure 2B, which manifested the dynamical changes in the expression levels of the cspC gene. The transitions between the low and high expression states over time were observed. After collecting the 69 real‐time trajectories including 6129 intensity data points under 0.9 mm Cm treatment, we generated a statistical histogram (Figure 2C, depicted by the blue bars) that clearly indicated two fluorescence intensity peaks, corresponding to the low and the high expression states of the cspC gene, within the cell population. Subsequently, a two‐state Continuous Gaussian Hidden Markov Model (CHMM) was employed to fit the fluorescence intensity data across these 69 real‐time trajectories, elucidating the transition dynamics between the observed bright and dim states.

HMM, an established probabilistic framework for analyzing time series data, serves as the foundation for this methodology. It can delineate the process of generating a sequence of directly unobservable (“hidden”) states within a Markov chain and subsequently deriving an observable output sequence from this state sequence. The HMM, inherently encompassing multiple hidden states, can facilitate the dynamical transitions of observational outputs, which can either shift between states or persist within a single state in response to temporal evolution. The assignment of the observations to respective states can be characterized by a probability density function (PDF), with each observation vector bearing an associated probability distribution across the various states. Within the context of the CHMM, a Gaussian PDF is employed. The parameters governing this Gaussian distribution, namely the mean matrix and covariance matrix, are determined via iterative optimization, a process integral to the Baum–Welch algorithm, which is specifically tailored for resolving the HMM training problem. Comprehensive details concerning this statistical analysis can be found in the dedicated *Statistical Analysis* Section.

Our CHMM fitting analysis unveiled the output probability distributions (as exemplified by the red, green, and black curves in Figure 2C), the residence time, transition probabilities, and instantaneous transition rates governing the two distinct fluorescence intensity states under 0.9 mm Cm treatment. The intensity centers (mean value of the Gaussian PDFs) were 108.64 and 121.34 RFU for the dim and bright states, respectively. The residence time of a state was defined as the weighted mean occupancy time of that state among all trajectories under a certain concentration of Cm induction. Normalized data indicated that the dim state occupied 58.7% (11.05 h) of the total observing time, while the bright state occupied 41.30% (7.77 h). The transition probabilities and transition rates between the two states are listed in **Table** **1**. Within the 12‐min observation time interval, cells residing in the dim state exhibited a transition to the bright state with a probability of 0.0209, while conversely, cells in the bright state manifested a probability of 0.0047 for transitioning to the dim state (as detailed in Row 4 of Table 1).

**Table 1:** The mean average fluorescence intensity values, state residence time, transition probabilities, and instantaneous transition rates of the two gene expression states (denoted as the “Dim” state and the “Bright” state) induced by increasing concentrations of Cm (from 0.6 to 0.9 mm), obtained from CHMM analysis. The transition probabilities and transition rates were calculated over 12‐min time intervals.

	Mean Average Intensity [RFU]		Residence time [%]		Transition probability		Transition Rate [1 min^−1^]	
	Dim	Bright	Dim	Bright	Dim to bright	Bright to dim	dim	Bright
0.6 mm	134.90	173.78	42.47 (5.44 h)	57.53 (7.36 h)	0.0209	0.0047	0.0018	0.0004
0.7 mm	120.22	157.76	45.91 (8.86 h)	54.09 (10.44 h)	0.0692	0.0080	0.0060	0.0007
0.8 mm	109.01	148.25	46.86 (6.82 h)	53.14 (7.74 h)	0.0526	0.0036	0.0045	0.0003
0.9 mm	108.64	121.34	58.70 (11.05 h)	41.30 (7.77 h)	0.0091	0.0047	0.0008	0.0004

For comparative purposes, we examined the kinetic parameters of the fluorescence intensity bistability induced by different Cm concentrations (0.6, 0.7, 0.8, and 0.9 mm). As outlined in the first column of Table 1, we observed that both of the states gradually became dimmer as the antibiotic concentration increased, with the mean average intensity values of the dim state decreasing from 134.9 to 108.64 RFU and those of the bright state from 173.78 to 121.34 RFU. Additionally, the residence time percentage for the bright state shortened from 57.53% (7.36 h) to 41.30% (7.77 h), while that of the dim state prolonged from 42.47% (5.44 h) to 58.70% (11.05 h) of the corresponding observing time.

### 2.3 Extending Gene Expression Bistability to Growth Rate and Cell Morphology Heterogeneities

**Figure 3:**
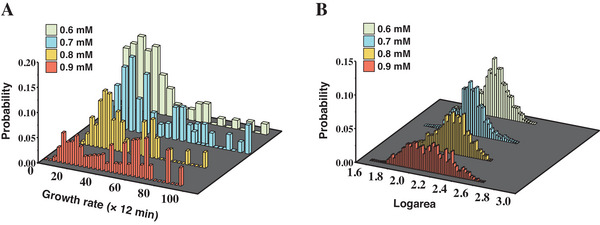
The distributions of growth rate (quantified by cell cycle time) and cell morphology (quantified by its area) under increasing Cm concentration induction from 0.6 to 0.9 mm. A) The growth rate distribution histograms at each Cm concentration were obtained by dividing the single‐cell real‐time trajectories into cell cycles based on the cell division events. More than 60 trajectories were analyzed in each Cm concentration. The microscope images were taken at intervals of 12 min. The methodology was presented in detail in the Experimental Section part. B) The log_10_ area distribution histogram was obtained from statistical analysis using the same trajectories.

As shown in Movies S1–S6 (Supporting Information), we noticed that a portion of cells within the same population exhibited not only differences in fluorescence intensity but also distinct cell morphology and growth rates. To accurately quantify these two cellular traits, we applied the same statistical methods for gene expression analysis to cell area (order parameter for morphology) and cycle times (order parameter for growth rate) using single‐cell real‐time trajectories. Our analysis results, depicted in **Figure** **3**, revealed that the growth rate also displayed bistability after induction by 0.6–0.9 mm of Cm, corroborating the findings of the prior research.^[^ ^6^ ^]^ Specifically, as the concentration of Cm increased, a peak representing the slow growth rate and longer division cycle emerged. Detailed quantitative data are listed in **Table** **2**, which shows that with increasing concentration of Cm, the overall growth rate was constrained. The mean cycle time values of the fast‐growing states prolonged from 20.20 (× 12 min) to 29.48 (× 12 min), while those of the slow‐growing states prolonged from 35.11 (× 12 min) to 73.29 (× 12 min). The residence times for the slow‐growing cells with longer cell cycle times also tended to increase. Furthermore, the distribution of cell area displayed a tendency of becoming wider spread as the concentration of Cm increased, indicating a heightened heterogeneity.

**Table 2:** The mean cycle time values, residence time, transition probabilities, and instantaneous transition rates of the two growth rate states induced by 0.6, 0.7, 0.8, and 0.9 mm Cm (referred to as the “Short” cycle time state and “Long” cycle time state), obtained from CHMM analysis. The transition probabilities and transition rates were calculated over 12‐min time intervals.

	Mean Cycle time [× 12 min]		Residence time [%]		Transition probability		Transition Rate [1 min^−1^]	
	Short	Long	Short	Long	Short to long	Long to short	short	Long
0.6 mm	20.1928	35.1071	80.07 (10.25 h)	19.93 (2.55 h)	0.0332	0.0048	0.0028	0.0004
0.7 mm	19.1218	61.5464	43.92 (8.94 h)	56.08 (11.42 h)	0.0161	0.0075	0.0014	0.0006
0.8 mm	23.0601	53.3791	55.97 (8.15 h)	44.03 (6.41 h)	0.0088	0.0048	0.0007	0.0004
0.9 mm	29.4895	73.2950	34.33 (6.56 h)	65.67 (12.25 h)	0.0120	0.0017	0.0010	0.0001

Notably, according to previous research, the Cm‐CAT feedback loop in our strain was expected to trigger a dormant state after antibiotic induction under the used experimental conditions.^[^ ^6^ ^]^ As shown in Movie S3 (Supporting Information), the cells in the field remained intact but non‐growing for ≈30 h. Meanwhile, the traced gene expression stayed at a low level. Concerns were raised that this low expression and non‐growing state might lead to cell death. To refute this conjecture, the dim cells with a slower growth rate after Cm induction were subjected to a low concentration of Cm to investigate if they could recover and resume growth. As shown in Figure S2 (Supporting Information), under 0.9 mm Cm induction, the selected cell gradually darkened in 12 h. After 13 h, the culture was switched to a normal antibiotic‐free medium. The cell brightened by degrees and started dividing again (Figure S2, Supporting Information), indicating that the bacteria cells under this fate were not dead. This behavior, observed in the sub‐MIC Cm culture medium, aligned with previous reports.^[^ ^6^ ^]^ Our system displayed a shorter recovery time, presumably due to the lower concentration of Cm to which the cells were switched.

### 2.4 Extending 1D Phenotypic Heterogeneities to Multidimensional by Trait Combination

**Figure 4:**
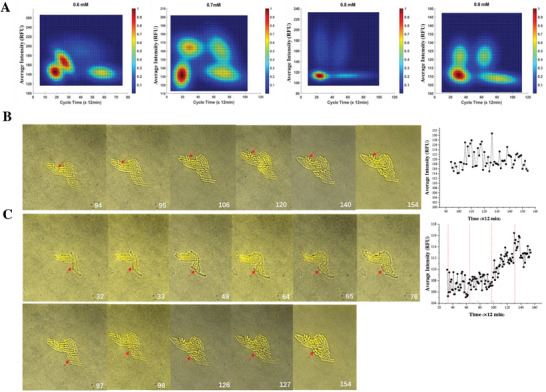
Four distinct and interchangeable states emerged after correlating fluorescence intensity and growth rate. A) CHMM‐fitted probability distributions after correlating the cspC gene expression (quantified by the reporter‘s average YFP fluorescence intensity) and the growth rate (quantified by cell cycle time) at Cm concentrations increasing from 0.6 to 0.9 mm, respectively. B) Representative images and fluorescence intensity trajectories indicated that the bacterial cell (red arrow) was kept intact for 60(× 12 min)with high intensity, noted as [L‐CT, H‐FL]. C) Another set of representative images and corresponding fluorescence intensity trajectories indicated that the cell (red arrow) divided three times and remained at a low fluorescence level during 98(× 12 min)of the recording process, noted as [S‐CT, L‐FL]. Then the cell switched into [S‐CT, H‐FL].

It was well established that the growth rate of bacteria can directly impact the metabolism activity^[^ ^11^, ^39^ ^]^ and gene expression.^[^ ^24^ ^]^ To elucidate potential correlations between the growth rate, cspC expression, and bacterial morphology, we collected and analyzed comprehensive datasets consisting of single‐cell cycle time, fluorescence intensity, and cell area trajectories from multiple single colonies exposed to varying concentrations of Cm. By evaluating two observables concurrently, we generated three distinct landscapes for the bacteria population under each Cm concentration. Specifically, the bimodality within the cell cycle time and average fluorescence intensity distributions directed our attention to their combined landscape. Within the specific biological context of our experimental investigation, the correlation of the two two‐state 1D datasets led to the possibility of delineating between two to four states within the 2D state space. According to our experimental observations under 0.6, 0.7, 0.8, and 0.9 mm concentrations of Cm treatment, the bacterial cells exhibiting either dim or bright fluorescence can possess a fast‐growing subpopulation and a slow‐growing subpopulation simultaneously. To visualize the 2D data across the four Cm concentrations, we plotted Gaussian Kernel Density Estimation (KDE) maps, as illustrated in Figure S3 (Supporting Information). The density aggregation appears to be distributed along both diagonal directions, resulting in the formation of a rectangular shape. To ascertain the appropriate number of states, we conducted K‐means clustering^[^ ^40^ ^]^ on the cycle time‐average intensity data and determined the optimal value for K (i.e., the number of states) using the elbow method.^[^ ^41^ ^]^ Upon comprehensive evaluation across the four Cm concentrations (0.6, 0.7, 0.8, 0.9 mm), our analysis revealed that the optimal K value, applicable across all concentrations, is four (refer to Figure S4, Supporting Information). This result resonated with our experimental findings. Subsequently, we employed a four‐state CHMM to fit the cycle time‐average intensity data. Figure S5 (Supporting Information) offers a comparative display of the K‐means‐clustered and CHMM‐fitted cycle time‐average intensity data points. In general, the CHMM fitting yielded a distribution that bore a closer resemblance to the KDE maps and exhibited better consistency in the positioning of the four states across all Cm concentrations. **Figure** **4**A depicts the probability distribution of the four states upon CHMM fitting, aligning not only with our experimental observations but also closely mirroring the density distributions observed in the KDE maps. Collectively, our experimental and statistical evidence supports the presence of four distinct states within our biological system.

We sought to explore this four‐state heterogeneity by comparing the behaviors of bacteria under 0.6, 0.7, 0.8, and 0.9 mm concentrations of Cm. As the concentration of Cm increased, the total percentage of residence time for bacteria displaying dim fluorescence with either fast or slow growth rate increased. As reported in the right columns of **Table** **3**, bacterial cells with low fluorescence intensity occupied 32.77% (4.22 h), 52.41% (10.71 h), 53.26% (7.91 h), and 62.18% (11.77 h) of the corresponding observing time for 0.6, 0.7, 0.8, and 0.9 mm Cm concentration, respectively. These results were consistent with those of 1D fluorescence intensity analysis. Furthermore, the proportion of bacteria exhibiting dim fluorescence and slower growth rate demonstrated a discernable increase as the concentration of Cm increased. For example, following exposure to 0.9 mm of Cm, the bacterial population was observed to segregate into four subpopulations, as previously mentioned and depicted in Figure 4A. The subpopulation is characterized by a long cycle time and low fluorescence intensity, denoted as the [L‐CT, L‐FL] state, centered at (81.15 (× 12 min), 108.28 (RFU)) in the landscape. According to the residence time given in Table 3, the [L‐CT, L‐FL] state of the cells occupied the largest percentage of observation time (38.55%, 7.30 h) both among the four states under 0.9 mm Cm induction and as compared to the same state in the other three Cm concentration groups. Conversely, the percentage of residence time for the cells with a short cycle time and high fluorescence intensity, referred to as the [S‐CT, H‐FL] state, decreased with increasing Cm concentration. Representative images and corresponding graphs are presented in Figure 4B,C, which clearly illustrate bacteria with both high and low levels of gene expression could hijack fast growth rates. Furthermore, an overarching trend was observed, whereby the corresponding mean values of the cycle times and fluorescence intensities of each state prolonged and decreased, respectively, with increasing Cm concentrations. This trend was also in consistency with our former 1D analysis results. For instance, in the 0.9 mm Cm induction group, the mean values of the [L‐CT, L‐FL] state were [81.15(× 12 min), 108.28 (RFU)], which were markedly longer in cycle time and lower in fluorescence intensity as compared to the corresponding values of [57.10 (× 12 min), 141.43 (RFU)] in the 0.6 mm group.

**Table 3:** The mean values and corresponding residence time of the four states emerging from cycle time and average fluorescence intensity correlation, calculated by CHMM via analyzing real‐time data.

	State mean value (Cycle time [× 12 min], average intensity [RFU])				Residence time [%]				
	S‐CT, L‐FL	S‐CT, H‐FL	L‐CT, H‐FL	L‐CT, L‐FL	S‐CT, L‐FL	S‐CT, H‐FL	L‐CT, H‐FL		L‐CT, L‐FL
0.6 mm	17.85, 143.53	25.78, 164.80	39.31, 187.52	57.10, 141.43	21.73 (2.80 h)	36.92 (4.68 h)		30.91 (3.98 h)	11.04 (1.42 h)
0.7 mm	18.15, 130.83	28.12, 163.27	67.56, 160.60	70.01, 132.55	23.56 (4.82 h)	30.09 (6.15 h)		17.50 (3.58 h)	28.85 (5.89 h)
0.8 mm	22.65, 111.89	24.98165.55	49.40, 158.61	57.86, 112.07	35.09 (5.25 h)	29.09 (4.25 h)		16.84 (2.46 h)	18.17 (2.66 h)
0.9 mm	29.76, 110.06	31.00, 121.25	62.77, 121.32	81.15, 108.27	23.63 (4.47 h)	13.37 (2.53 h)		24.45 (4.63 h)	38.55 (7.30 h)

**Table** **4**a–d lists the transition probabilities between the four states for each Cm concentration group, ascertained through CHMM analysis. Each state could either remain the same or undergo three possible transitions to other states. For example, under 0.9 mm Cm induction, the [S‐CT, L‐FL] state exhibited a probability of 0.013 to switch into [S‐CT, H‐FL], 0.0046 to switch into [L‐CT, H‐FL] and 0.0094 to switch into [L‐CT, L‐FL] state. However, it was more difficult for the three states to revert back to their initial state, with probabilities in the order of 10^−4^ for [L‐CT, H‐FL] and [L‐CT, L‐FL], and 0.0044 for [S‐CT, H‐FL]. Furthermore, the [S‐CT, H‐FL] state might also switch into [L‐CT, H‐FL] with a probability of 0.0077, and the latter state 0.0030 to switch back. For the [L‐CT, L‐FL] state, it appeared to switch into [L‐CT, H‐FL] with a likelihood of 0.0036, which was the highest probability among its three transition routes. The corresponding instantaneous transition rates of the four states under different concentrations of Cm treatment are presented in Table 4 e.

**Table 4:** The transition probabilities and instantaneous transition rates (1 min^−1^) among the four states at different concentrations. The transition probabilities and transition rates were calculated over 12‐min time intervals. Each element in the transition probability tables refers to the transition probability from the row state to the column state.

a) Transition probability of the four states under 0.9 mm Cm treatment.				
0.9 mm	[Cycle time [× 12 min], Average fluorescence intensity [RFU]]			
	[S‐CT, L‐FL]	[S‐CT, H‐FL]	[L‐CT, H‐FL]	[L‐CT, L‐FL]
[S‐CT, L‐FL]	0.9730	0.0130	0.0046	0.0094
[S‐CT, H‐FL]	0.0044	0.9879	0.0077	5.5735 × 10^−13^
[L‐CT, H‐FL]	7.5351 × 10^−4^	0.0030	0.9962	1.3925 × 10^−7^
[L‐CT, L‐FL]	5.0443 × 10^−4^	2.0458 × 10^−11^	0.0036	0.9958

b) Transition probability of the four states under 0.8 mm Cm treatment.				
0.8 mm	[Cycle time [×12 min], Average fluorescence intensity [RFU]]			
	[S‐CT, L‐FL]	[S‐CT, H‐FL]	[L‐CT, H‐FL]	[L‐CT, L‐FL]
[S‐CT, L‐FL]	0.9753	0.0188	6.9640 × 10^−4^	0.0052
[S‐CT, H‐FL]	7.8470 × 10^−26^	0.9966	0.0034	5.9701 × 10^−41^
[L‐CT, H‐FL]	2.1296 × 10^−25^	8.0179 × 10^−15^	1.0000	2.4373 × 10^−27^
[L‐CT, L‐FL]	0.0057	1.5618 × 10^−33^	0.0164	0.9779

c) Transition probability of the four states under 0.7 mm Cm treatment.				
0.7 mm	[Cycle time [× 12 min], Average fluorescence intensity [RFU]]			
	[S‐CT, L‐FL]	[S‐CT, H‐FL]	[L‐CT, H‐FL]	[L‐CT, L‐FL]
[S‐CT, L‐FL]	0.9679	0.0193	1.7039 × 10^−16^	0.0128
[S‐CT, H‐FL]	2.6074 × 10^−14^	0.9945	0.0055	1.8204 × 10^−6^
[L‐CT, H‐FL]	0.0055	0.0075	0.9801	0.0069
[L‐CT, L‐FL]	1.9712 × 10^−13^	0.0012	0.0152	0.9836

d) Transition probability of the four states under 0.6 mm Cm treatment.				
0.6 mm	[Cycle time [×12 min], Average fluorescence intensity [RFU]]			
	[S‐CT, L‐FL]	[S‐CT, H‐FL]	[L‐CT, H‐FL]	[L‐CT, L‐FL]
[S‐CT, L‐FL]	0.9522	0.0402	3.9669 × 10^−9^	0.0077
[S‐CT, H‐FL]	0.0012	0.9762	0.0226	1.2595 × 10^−34^
[L‐CT, H‐FL]	8.5961 × 10^−12^	0.0055	0.9937	7.2868 × 10^−4^
[L‐CT, L‐FL]	3.3588 × 10^−68^	9.5144 × 10^−32^	0.0080	0.9920

e) Instantaneous Transition rates [1 min^−1^] of the four states under four concentrations of Cm treatment.				
	[S‐CT, L‐FL]	[S‐CT, H‐FL]	[L‐CT, H‐FL]	[L‐CT, L‐FL]
0.6 mm	0.0041	0.0020	0.0005	0.0007
0.7 mm	0.0027	0.0005	0.0017	0.0014
0.8 mm	0.0021	0.0003	6.6613 × 10^−16^	0.0019
0.9 mm	0.0023	0.0010	0.0003	0.0004

From Table 4, we can calculate the overall probability of transitions occurring between the S‐CT states ([S‐CT, L‐FL] and [S‐CT, H‐FL]) and the L‐CT states ([L‐CT, L‐FL] and [L‐CT, L‐FL]), as well as between the L‐FL states ([S‐CT, L‐FL] and [L‐CT, L‐FL]) and the H‐FL states ([S‐CT, H‐FL] and [L‐CT, H‐FL]) at each Cm concentration. By comparing the total transition probabilities among the four states characterized by two cellular traits with those characterized by a single trait (either the cell cycle time or the cspC gene expression), we can illuminate the potential correlation between these two dimensions of cellular trait. For instance, under 0.9 mm Cm concentration treatment, the total probability of transitioning from the [S‐CT, L‐FL] or [L‐CT, L‐FL] state to the [S‐CT, H‐FL] and [L‐CT, H‐FL] states was 0.0212. In contrast, the transition probability from the Dim state to the Bright state was 0.0091, representing the hypothetical total transition probability if the transitions between the four states are entirely independent. Table S1 (Supporting Information) provides a comparative overview of the transition probabilities between states characterized by one or two cellular traits across different Cm concentrations, illustrating consistent disparities in all state transitions or Cm concentrations. This substantiates the relevance of the two cellular traits.

**Figure 5:**
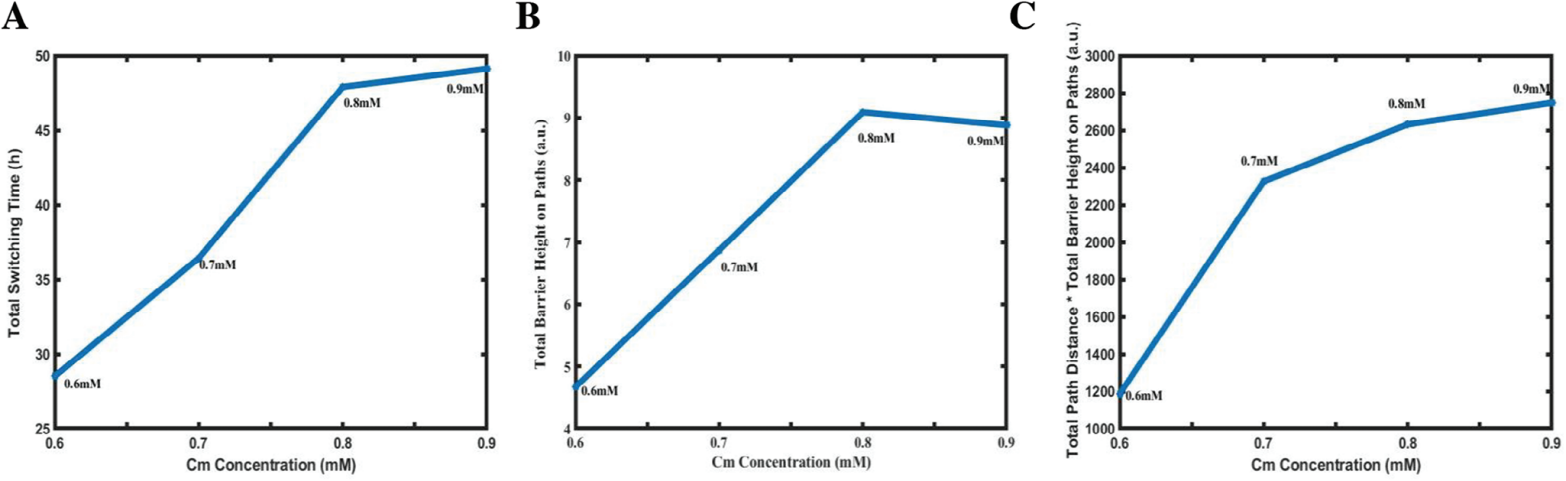
A) Total state switching time, B) Total barrier height on the switching paths, and C) Total switching path distance × total barrier height on the paths of the four states emerging from growth rate and fluorescence intensity correlation versus different Cm concentrations.

While the transition probabilities revealed the likelihood of state changes, switching time, also obtained from CHMM analysis, could provide information on the actual time spent when a state switched. Specifically, the switching time is defined as the average duration of time a bacterial cell remains in its initial state before transitioning to the terminal state across all Hidden Markov Chains derived from the same group of experimental data. Table S2 (Supporting Information) presents the switching time for each state at four different Cm concentrations. Notably, consistent with the small value elements in transition probability matrixes, some transitions were not observed in the CHMM‐fitted trajectories, and therefore, no switching paths or switching times were assigned to these transitions. We defined the number of switching paths as the count of observed state transitions among the four states within the trajectories of each Cm concentration. For instance, by mapping states 1, 2, 3, and 4 to the respective descriptions of [S‐CT, L‐FL], [S‐CT, H‐FL], [L‐CT, H‐FL], and [L‐CT, L‐FL], it was discerned that, at a Cm concentration of 0.9 mm, there were no transitions between state pairs 2 and 4, 3 and 4, or 4 and 2. This absence of transitions gave rise to a total of nine switching pathways. Similarly, at 0.8 mm Cm, there were no observed transition behaviors from state 2 to 1, state 2 to 4, state 3 to 1, state 3 to 2, state 3 to 4, or state 4 to 2, leading to six switching paths. The maximum number of switching paths was nine at 0.9 mm Cm, while the minimum number was six at 0.6 mm and 0.8 mm Cm (Figure S6A, Supporting Information). We also calculated the total switching time by summing the switching time of all possible switching paths among the four states, which reflected the overall difficulty level of state change. As shown in **Figure** **5**A, the total switching time increased with increasing concentrations of Cm, indicating that higher concentrations of Cm impede the ability of bacterial cells to alter their biological state.

In addition, we investigated the distributions under different Cm concentrations after associating cell area with growth rate or average fluorescence intensity. The distributions remained bistable because the area showed nearly mono‐stability and it would not significantly affect the distributions after the correlations. We present the quantitative results of these two correlations in Table S3 (Supporting Information). Our analysis revealed that the bacterial cells with smaller areas tended to exhibit higher fluorescence intensity, while those with larger areas tended to be dimmer. This observation was in line with the role of the dilution effect in such systems.^[^ ^16^ ^]^ Furthermore, we observed a marked shift in cell size as the concentration of Cm increased: the bacterial cells with higher fluorescence intensity tended to have smaller areas, while those with lower intensity appeared to have larger areas compared to the cells treated with lower Cm concentrations. We also found that the smaller cells tended to have shorter cycle times and the larger cells longer cycle times (see Table S4, Supporting Information). Moreover, as the concentration of Cm increased, the cycle times for all cells became longer, and the cell areas became larger for the cells with longer cycle times, Overall, our findings suggested that while the addition of antibiotics could promote cell morphology heterogeneity, it did not appear to generate statistical bistability in cell size, at least not in our experiments.

### 2.5 Dynamical and Thermodynamic Origins of Multi‐Dimensional Phenotypic Heterogeneity

To explicate the dynamical and thermodynamic origins of this heterogeneous behavior arising from the cycle time and average fluorescence intensity correlation, we applied the non‐equilibrium landscape and flux theory for analysis. The dynamics in biological systems, such as the bacterial state‐switching behaviors characterized by the switching paths and switching time, are determined by the population‐potential landscape and the steady‐state probability flux. With the knowledge of steady‐state probability distribution and transition matrix, we can decompose the dynamics into a detailed balance‐preserving landscape part and a detailed balance‐breaking flux part, quantifying the potential landscape with basins denoting low potential or high probabilities and the flux landscape consisting of multiple flux loops.^[^ ^36^ ^]^ We quantify the potential landscape as the negative natural logarithm of the steady‐state probability distribution, a distribution derived directly from the output probabilities obtained through CHMM fitting. The landscape serves as a surrogate for quantifying the weight of various cell fates. It can provide a global characterization and a stability measure of the bacterial population in terms of the depth of the state basin of attraction.^[^ ^42^ ^]^ In parallel, we define the flux as the net local steady‐state probability flux between two states, encapsulating the transition probability flow among distinct states within the biological system. The steady‐state probability flux, constituting the dynamical origin of nonequilibrium, can reshape the landscape and influence the probability of the cell fates.

Environmental perturbations can have discernible effects on the stability of biological systems, which will manifest through observable quantities such as the switching time. Furthermore, these perturbations can also influence the potential landscape and the probability flux. Leveraging the non‐equilibrium landscape and flux theory, we can unveil the intricate interplay between the experimental observations and the non‐equilibrium dynamics. We first obtained the most probable routes of all existing switching paths, as well as the barrier heights on those routes, from the potential landscapes of each Cm concentration (presented in Figure S7, Supporting Information). The most probable switching route referred to the one with the shortest distance between the valley points of the two states on the landscape, and the barrier height was the height difference between the highest point en route and the starting point or destination point on the landscape. The switching routes and the barrier heights indicated the degree of difficulty associated with the bacterial state‐switching behavior, consequently having an influence on the switching time. The change of total switching path distance and total barrier height with respect to antibiotic concentrations were plotted in Figure 5B and Figure S6B (Supporting Information). If we envision the bacterial state as a ball rolling on the landscape, the switching time required for it to move from one basin to another is determined by both the length of the route and the barrier height between basins. Guided by this conception, we calculated the product of total route distance and total barrier height under different antibiotic concentrations and found that the results exhibited a tendency matching that of the total switching time (Figure 5C). This finding signified that in a potential landscape, the total distance and barrier height indeed collectively determined the overall degree of difficulty associated with state‐switching, and consequently, the total switching time. Furthermore, it indicated that the potential landscapes could provide insights into the root cause of observable biological behaviors.

We then delved deeper into the underlying basis from perspectives of nonequilibrium dynamical driving force as average flux value, thermodynamic cost as entropy production rate (EPR), and time irreversibility of cross‐correlation. The steady‐state probability flux, being rotational due to the probability conservation law at the long time limit, can reveal the tendency of flow among states, rather than being localized in a specific state. Therefore, the flux can be used to quantify the degree of the state instability within the bacterial population and can influence the distribution of the landscape under different conditions. A non‐zero flux indicates that there is a net probability input or output from or to the system, facilitating the transitions between different cell fates and influencing the transition probabilities. While the flux landscape can quantify the degree of detailed balance breaking (non‐zero net flux flow) and constitutes the dynamical origin of state instability and time irreversibility, EPR is the thermodynamic cost dissipated to maintain the steady state. The physical meaning of the EPR is the total entropy production of both the system and its environments, being greater than or equal to zero. Similar to how electric circuits can generate heat as a consequence of voltage (potential) and current (flux), the landscape and flux can globally determine the production of entropy. Specifically, the flux serves as the origin of entropy production because the mathematical expression of EPR is directly related to the flux, implying that the EPR can also reflect the state instability characterizing the bacterial population from the thermodynamic perspective.

**Figure 6:**
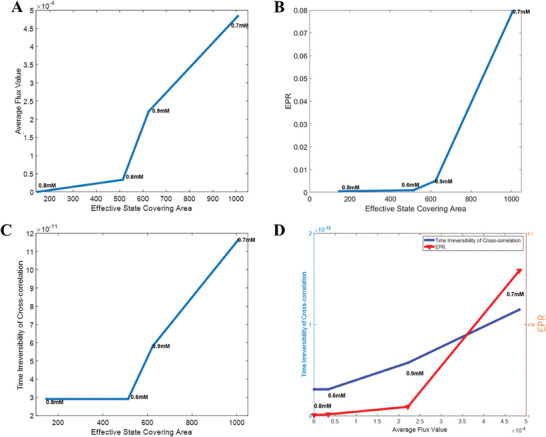
The change of the effective state covering area, average flux loop value, the EPR, and the time irreversibility of cross‐correlation with respect to the variations of Cm concentration. A) Average flux loop value, B) entropy production rate (EPR), and C) time irreversibility of cross‐correlation versus effective state covering area upon variations of Cm concentrations (0.6, 0.7, 0.8, and 0.9 mm Cm). D) Comparison of EPR and time irreversibility of cross‐correlation versus average flux value at different concentrations of Cm.

In biological systems, altering culture conditions will initiate shifts in the flux, subsequently reshaping the landscape and influencing the EPR as well as the time irreversibility. The reshaping of the landscape was manifested in the significance degree of the four states, that is, how distinct or prominent the states were. The significance degree of the states can be quantified by the effective state covering area, which was defined as the x‐y plane projection area with probabilities higher than 0.2 in the potential landscape. It signified the overall likelihood of bacteria to assume the cell fates under a certain condition. Conversely, a landscape with a more distinct and spread‐out state distribution was likely to generally encompass greater transition probabilities. This, in turn, translated to higher values of the flux. As shown in **Figure** **6**A–C, the variation trends of the average flux value, EPR, and the time irreversibility of cross‐correlation exhibited remarkable similarity with the variations in the significance degree of the four states under different Cm concentrations. According to Figure 4A, two out of four states were distinct in 0.8 mm, and three were distinct in 0.6 mm. In 0.9 mm, four distinct states appeared, while in 0.7 mm, the four distinct states became more prominent and separated. When interpreting these trends in terms of the variation of physical quantities, it became evident that the average flux, EPR, and time irreversibility increased as the states became more distinct and their corresponding effective covering areas expanded. 0.7 mm of Cm yielded the highest values for the average flux, EPR, and time irreversibility. By contrast, the landscapes with two and three distinct states were less extensive and exhibited lower average flux value, EPR, and time irreversibility of cross‐correlation relative to those with four distinct states. Furthermore, as depicted in Figure 6D, the variation of the EPR and the time irreversibility of cross‐correlation with respect to the average flux value also exhibited a similar pattern, highlighting that the origin of the EPR and the time irreversibility lies in the flux. These quantitative results underscored the role of nonequilibrium driving force flux in redistributing the landscapes and elevating the time irreversibility, while the EPR measured the thermodynamic cost entailed during the process. Therefore, the difference in the significance degree and stability of the four states under different concentrations of Cm could be described and explained.

On the other hand, the tendencies of average flux value, EPR, and time‐irreversibility of the cross‐correlation with respect to changes in Cm concentration closely resembled that of the total switching path distance (Figure S6B, Supporting Information). This demonstrated another effect that the flux had on the landscape. Specifically, the probability flux loops flowing in the state space tended to destabilize the current state and generate new states, thereby providing the dynamical origin of the instability of the current state and the emergence of new states. EPR provided the thermodynamical cost associated with the new state emergence, thus offering the thermodynamic origin for the instability of the current state and the emergence of new states. Hence, they increased the travel distance in the landscapes during state switching. Consequently, a longer switching distance would also increase the time irreversibility. However, the variations of the flux value and the total switching path distance are not identical. The reason behind this is that, while the flux value was calculated by decomposition of the transition probability matrix which had no zero elements, the total path distance was based solely on the observed switching paths. Though some switching paths were not observed in the HMM trajectories and did not contribute to the total distance, they possessed a positive value in the transition probability matrix and constituted the flux loops.

These non‐equilibrium dynamical and thermodynamic quantifications of the flux and EPR expanded the scope of our experimental findings, offering valuable physical insights into the dynamic phenotypic heterogeneity in resistant *E. coli* under antibiotic stress. In particular, these results can help us understand the intricate mechanisms that govern the cell fate potentials and switching kinetics of the bacteria. The subsequent Discussion Section delves into the broader biological implications of our findings.

## 3 Discussion

As previously reported, the impact of Cm on growth rate manifested globally in a passive manner.^[^ ^37^ ^]^ Under such conditions of translation‐limited growth, the expression levels, or the protein concentrations, of the unregulated genes decreased linearly with the decreasing growth rate. This behavior could likely be the generic consequences of ribosome activities upon translational inhibition, rather than the relatively weak dilution or accumulative effect of the growth rate.^[^ ^6^, ^21^, ^23^ ^]^ A faster growth rate corresponded to less ribosomal inhibition, which in turn rendered the gene expression level higher. In this case, when there was a growth rate bistability, the growth rate and the ribosome activity led to two gene expression states, with the gene expression level being proportional to the growth rate. However, our investigation revealed two unexpected states, whereby the cells with a high growth rate displayed low gene expression and those with a low growth rate displayed high gene expression.

**Figure 7:**
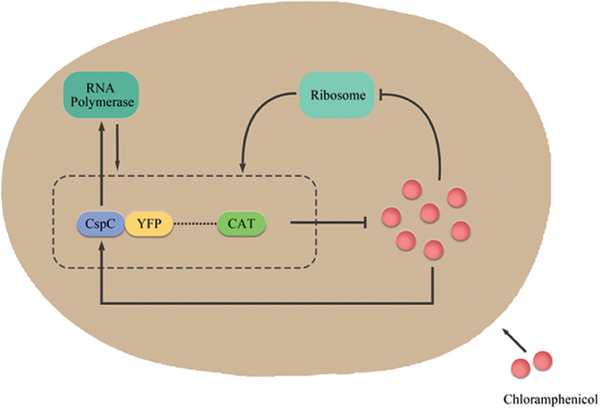
Simplified schematic diagram of mechanisms behind the emergence of the four phenotypes. The interaction between Cm and the CAT expressed by the Cm‐resistance enzyme gene located in the *E. coli* chromosome constitutes a positive feedback loop. As one of the stress response genes,^[^ ^27^ ^]^ cspC can be induced by Cm and up‐regulate the RNA polymerase,^[^ ^29^ ^]^ which promotes transcription of global genes and thus constitutes a second positive feedback loop. YFP reporter is inserted in the promoter controlling the expression of cspC.

To understand this counterintuitive four‐state heterogeneous behavior, we examined the selected target gene cpsC. It has been reported that, in addition to temperature stressors, antibiotics were capable of more strongly inducing the cspC gene.^[^ ^27^, ^28^, ^29^, ^43^ ^]^ We arrived at a simple gene‐expression network model, as **Figure** **7** indicated, the interplay between Cm and the cspC gene could constitute a positive feedback loop. Our observed behaviors supported the notion that the growth rate and Cm jointly regulated the cpsC gene expression. Indeed, the addition of Cm not only exerted a regulatory effect on the growth rate but also directly influenced the expression of cspC gene, resulting in a deviation from the anticipated linear response between expression and fitness. With this in mind, overlaying the effects of the Cm‐CAT and Cm‐cspC feedback loops, the enhanced heterogeneity of gene expression and growth rate in our experiment could be elucidated. Specifically, the cells incapable of producing an adequate quantity of CAT protein to degrade Cm exhibited sluggish growth rates and subdued gene expression levels. However, due to the interplay between Cm and our target gene cspC, inadequate CAT production led to copious Cm accumulation, which in turn promoted cspC expression and RpoS, resulting in a state of languid growth but elevated expression.^[^ ^44^ ^]^ Conversely, the cells capable of producing sufficient CAT protein to suppress Cm tended to display accelerated growth rates as well as elevated gene expression levels. Nevertheless, the expression level of cspC gene was comparatively subdued upon increased Cm degradation, resulting in a state with a high growth rate but low gene expression level. Therefore, we suggest that the gene feedback loops and the growth rate bistability gave rise to four states together. Additionally, investigations can be conducted on RNA polymerase‐related genes such as the rpoN gene^[^ ^29^ ^]^ or other genes to corroborate these findings.

Among the four states of bacteria under sub‐MIC concentrations of Cm treatment, we could analogize the two subpopulations with insufficient CAT and slower growth rates as persisters. Though not persisters by definition, these cells possessed persister‐like characteristics such as Cm tolerant, slow‐growing, and viability following the removal of antibiotics.^[^ ^45^, ^46^, ^47^, ^48^ ^]^ The other two states of bacteria that produced sufficient CAT protein and displayed high growth rates were inherently resistant cells. This dichotomy of behavior could be explained by the “division of labor” strategy employed by the bacterial populations. In this strategy, the persister‐like subpopulation remained inactive in growth and functioned as seeds ready to germinate when a suitable environment emerged, while the resistant subpopulation kept a rather fast reproduction rate and sustained continuous infection. Within the growth‐retarded persister‐like subpopulation, cells exhibited both low and high gene expression levels, indicating that persistence could be maintained when cells were not completely dormant. The persister‐like cells with active metabolism might have adopted the tactic of producing various proteins to maintain basic metabolism and combat Cm invasion.^[^ ^49^ ^]^ This portion of the cells exhibited an interesting behavior that could be exploited in future studies. Meanwhile, the fast‐growing resistant cell subpopulation could display high expression, as previously reported, but those with suppressed expression were atypical. Over an extended period of time, we observed that this portion of the cells eventually went to lyse as Figure S8 (Supporting Information) indicated, or switched into highly expressed cells with short cell cycles (Figure 4C). Moreover, as the concentration of Cm increased, the portion of bacteria with a slow growth rate and low expression, corresponding to the persister‐like subpopulation, appeared to increase. Previous research has indicated phenotypic heterogeneity occurred prior to gene mutations which led to evolution.^[^ ^4^ ^]^ This suggested that persistence, as a multidrug‐tolerant and mostly dormant phenotype, might represent the initial response to ensure bacterial survival under harsh environmental conditions. With prolonged exposure to the same environment, single‐drug‐tolerant resistance might emerge as a subsequent response to ensure bacterial reproduction, proceeding with the evolution process.

Herein, we were able to observe the state‐switching behaviors of bacteria cells by the microscope. On the one hand, we found that under all concentrations of Cm treatment, the cells exhibited a high probability (>95%) of remaining in their original state across successive generations. This behavior resembled that of epigenetic inheritance. In our experiment, while the gene regulation pathways and growth rate collectively determined gene expression levels and phenotypes, the role of epigenetics in this process remained unknown and might propose an interesting topic for future research. On the other hand, as the concentration of Cm increased, the bacteria cells became even more reluctant to change their biological status. We endeavored to explain this dynamic behavior from the perspective of non‐equilibrium dynamics and thermodynamics, thereby establishing a quantitative relationship and providing a methodology that could guide the experiments and associated simulation. Specifically, the potential landscape and the flux landscape were two crucial features that governed the dynamics and global properties of the gene network. The difference between the average fluorescence intensity – cycle time landscapes under different Cm concentrations could be attributed to the change of flux loops flowing through the state space. The landscapes for bacteria exposed to different Cm concentrations underwent a process of redistribution which was regulated by the flux. As the flux increased, the system was prone to be less stable for the current state and tended to generate new states, thus increasing the effective state covering areas, the total distance of all possible switching paths, as well as the time‐irreversibility and entropy production rate. The switching behavior between different states was determined by two major elements: route length and barrier height, both of which could be quantified on a landscape. By correlating these two elements through multiplication, we identified a trend matching that of the total switching time under different concentrations of Cm. This suggested that as the concentration of Cm increased, bacteria cells spent a longer time on the state‐switching process, making it more difficult to convert into other states.

There was, however, a subtle inconsistency between the tendencies of cause and effect in the considered scenario, namely the flux value and total switching path distance. While their variation with respect to the Cm concentration was comparably similar, it was not entirely identical. This disparity might have stemmed from the fact that the complete set of state‐switching behaviors was not observed in our experiment. We could not trace all 12 switching paths in either of the four concentrations tested, owing to some extremely low transition probabilities and a limited volume of available data. Nevertheless, the CHMM fitting results gave us transition probability matrixes devoid of zero elements, suggesting the possibility of occurrence for all switching paths, albeit some being highly improbable. As a result, when we decomposed the transition probability matrix to obtain flux loops, all 12 possible switching circumstances contributed to the flux value. In contrast, only the switching that actually happened in our trajectories was included when calculating the total switching path distance.

It is worth mentioning that the statistical analysis in our investigation was based on the experimental observation that the bacterial cells subjected to sub‐MIC concentrations of Cm treatment can exhibit either fast or slow growth rates while concurrently possessing subpopulations with high and low gene expression levels. While we acknowledge that the confirmation of the four states via the elbow method of K‐means clustering included a degree of heuristic interpretation, the amalgamation of our experimental and statistical evidence adequately supported the presence of four‐state heterogeneity. Moreover, the four‐state CHMM fitting generated landscapes that closely mirrored our experimental data points. However, given the nature of this fitting method, the selection and distribution of the four states inherently involved certain subjectivity.

In addition, we noticed that phenotypic heterogeneities can emerge in microorganisms besides bacteria. Recently, increasing attention has been directed towards the role of the cancer cells with a drug‐tolerant persister (DTP) state. They are recalcitrant to cancer therapy and may lead to cancer recurrence.^[^ ^50^, ^51^ ^]^ Bacteria are often used as analogs due to their much less complexity and known mechanisms. Therefore, revealing the global phenotypic heterogeneity and the switching dynamics of the bacteria is also critical for understanding more about the DTP state cancer cells. Our new findings can provide quantitative information to identify the key biological processes related to antibiotic resistance and the DTP state cancer cells, thus helping to find potential solutions to control recurring infections and cancer.

## 4 Conclusion

In conclusion, we conducted experimental investigations to quantify bacterial cell fate decision‐making behaviors under the influence of translation‐inhabiting antibiotics. We observed multiple dimensions of phenotypic heterogeneity, including the gene expression, fitness, and morphology in the resistant *E. coli* strain when exposed to Cm. Our study expands on previous reports that have focused solely on individual observable parameters. In addition to the growth rate, we identified the bistability of the cspC gene expression implied by the growth behavior and quantified the observable dynamical behaviors. Remarkably, by establishing a correlation between expression and fitness, we identified four cell fates, two of which were unexpected. We proposed a simple gene expression network model and revealed the underlying process. Cm invaded the ribosome of the *E. coli* K‐12 strain and restrained its translation for a subset of the cells. This led to protein deficiency and restricted growth rates, while the rest of the cells expressed resistant CAT proteins that exceeded a certain threshold, sufficient for the degradation of the surrounding antibiotic, thereby enhancing the growth rate. As a result, growth rate bistability appeared, which had an impact on gene transcription. Cm also interacted with the cspC gene and induced up‐regulation of RNA polymerase, which further encouraged gene expression and vice versa. This caused the deviation of the bistability and generated four states after the gene expression and growth rate correlation. Thereupon, we were able to explain and extrapolate the biological significance of the four phenotypes and their role in microbial stress response and evolution. We also elucidated how landscape and flux collectively determined the non‐equilibrium dynamics and thermodynamics while influencing observable biological behaviors. The non‐equilibrium driving force flux was the dynamical origin of new state generation, destabilizing the landscape, generating elongated switching paths, and increasing time‐irreversibility as well as the thermodynamical cost. These intrinsic processes reshaped the landscape and altered the switching routes, resulting in different state‐switching behaviors. In this way, we quantitatively established the relationship between the physical quantities and the biological behaviors, which could provide a reference for experimental and simulation research. Furthermore, our results showed that a higher concentration of antibiotic treatment rendered the resistant bacteria more reluctant to change into other physiological states, which might provide insight for controlling repeated bacterial infection or cancer recurrence after treatment. Understanding the molecular basis and the individual dynamical process is crucial for effectively choosing the bacterial population when facing the existing treatment options and providing instruction to find effective solutions to control infections.

## 5 Experimental Section

### Strains, Culture, and Pre‐Measurement Disposure

The strains used in the paper were derivatives of *Escherichia coli (E. coli)* K‐12 which were bought from the *E. coli* Genetic Stock Center^[^ ^30^ ^]^ (Yale University, New Haven). The observed target gene was cspC, a member of cold shock proteins, with the function of up‐regulating the level of RNA polymerase. The CAT gene was located downstream of each YFP fusion behind its own constitutive promoter, the whole strain could be expressed as cspC‐YFP(::cat). The bacteria cells were first cultured in LB from a single colony picked on an agar plate for 6–7 h, then they were transferred into M9 minimal medium supplemented with glucose (0.4%) and amino acids (2%, 50 × MEM amino acid solution, Gibco) shaking at 250 rpm under 37 °C and cultured for overnight. Small gel pads with ≈2% agarose (Sigma) were prepared through heating and placed between the microslide and coverslip of the FSC2 to immobilize the cells from moving. Then 0.4 µL of the bacteria solution was injected into the pads. Fresh M9 minimal medium with different Cm concentrations was supplemented to the culture area in real‐time with a peristaltic pump. Cm stock solution (20 mg mL^−1^, Aladdin) dissolved in 70% isopropanol solution was prepared before dilution into 0.2–1.0 mm in M9 medium.

### Microscopy Measurements and Image Acquirement

A fluorescence inverted microscope system (Nikon Ti‐U) with an automated stage, shutters, and 100 × oil immersion objective was adopted. Fluorescence images were acquired by using an argon ion laser with 5 mW at 514 nm (the excitation wavelength of YFP) and the cooled EM‐CCD camera (iXon3EMDU‐897, Andor, Connecticut, USA). The whole FSC2 device (Bioptechs) was kept at 37 °C by equipping a temperature control device (Bioptechs) during the experiment. For the steady‐state observation and data collection, microscopic images of the bacterial cells cultured in batches were captured at multiple time stages (the 10th h, 20th h, and 30th h following treatment) for each Cm concentration. The dataset for each Cm concentration group at each time point comprised more than 2000 cells. For the real‐time observation and data collection, the cells (0.4 µL of the bacteria solution) were first transferred to the microfluidic chip of FSC2 and fixed onto a steerable microscope stage, and then they were cultured in M9 medium containing different concentrations of Cm at a speed of 1.0 rpm with LongerPump. 6–10 different positions for each concentration far away from other cells were selected to avoid further over‐crowding, and then they were monitored and captured in parallel every 12 min during a period of 30–35 h.

### Statistical Analysis—Microscopy Image Analysis

After collecting images with Metamorph, image analyzing was conducted by virtue of automated analysis and manually checked custom MATLAB code (Schnitzcells). The relative growth rate of the bacterial cells, as presented in Figure 1C, was determined by the number of generations a single‐cell underwent over a 30‐h period. Each dataset for a specific Cm concentration comprised of the relative growth rates obtained from 8 micro‐colonies.

### Statistical Analysis—Fitness, Fluorescence Intensity, and Area Real‐Time Trajectories Data Collection

The real‐time trajectory of each colony was tracked and analyzed according to previously reported methods and software (Matlab, R2018b, ww2.mathworks.cn, RRID:SCR_0 01622). A Matlab code was designed that allows fluorescence intensity and area extraction and data export by means of time trace for further statistical analysis. For individual colonies, spots with complete division cycles were collected for correlation calculation. The division events were recognized and labeled at corresponding time nodes for growth rate analysis. To better illustrate the growth rate distribution, the following method was adopted to balance the number fast division in a fixed period. Given a division time of 15, it was noted as 15 times of 15. And when the division time of 60, it was noted as 60 times of 60.

### Statistical Analysis—Dynamic Trajectories Analysis by CHMM

Using experimentally collected real‐time data of average fluorescence intensity, cell area, and cycle time under four different antibiotic concentrations, 1D, 2D, and 3D frequency distribution maps were plotted. Two cell states could be identified from the distribution graphs of both average fluorescence intensity and cycle time, while the cell area showed no significant sign of multiple states. a two‐state CHMM was used to fit the 1D average fluorescence intensity and the cell cycle time data, obtaining the transition dynamics between the states. Specifically, the normalization of the CHMM output probabilities was calculated by dividing it based on the maximum value within the group. When dealing with the 2D data, the elbow method was first employed based on K‐means clustering to confirm the number of cell states. Results showed that there were most likely two states with average fluorescence intensity and cell area or cycle time and cell area being the independent variables. When average fluorescence intensity and cycle time were the independent variables, however, four states were the most probable case.

To quantitatively distinguish cell states described by two traits and attain information about their initial probability, transition probability, switching rate, and residence time, a four‐state CHMM was applied to the 2D data sets. The aim was to take a set of experimentally observed sequences and acquire the best model parameters. To solve this HMM training problem, a MATLAB code was written based on the Baum–Welch algorithm and Viterbi algorithm. The iterative HMM analysis began with manually inputting random parameters, followed by iterations using the Baum–Welch algorithm and Viterbi algorithm to re‐estimate parameters, track switching behavior, and calculate output probabilities. A maximum likelihood estimation of the HMM parameters was performed on all the time trajectories separately. The analysis process would stop when the difference between the two last total output probabilities was less than the pre‐set value.

### Statistical Analysis—Non‐Equilibrium Dynamic and Thermodynamic Calculations

The calculations were executed using customized MATLAB packages that were developed based on specific definitions of the quantities and methodologies drawn from prior publications.^[^ ^36^, ^52^ ^]^

## Conflict of Interest

The authors declare no conflict of interest.

## Supporting information

Supporting Information

Supplemental Movie 1

Supplemental Movie 2

Supplemental Movie 3

Supplemental Movie 4

Supplemental Movie 5

Supplemental Movie 6

## Data Availability

The data that support the findings of this study are available from the corresponding author upon reasonable request.
