# Supplementary material for: Quantifying Dynamic Phenotypic Heterogeneity in Resistant Escherichia coli under Translation‐Inhibiting Antibiotics
Source: Adv Sci (Weinh). 2024 Jan 9;11(11):2304548. doi: 10.1002/advs.202304548 (PMC10953537; doi:10.1002/advs.202304548)
Supplement: Supplementary file 1 — Supporting Information [file ADVS-11-2304548-s006.pdf]

ADVANCED SCIENCE
Open Access

Supporting Information

for *Adv. Sci.*, DOI 10.1002/advs.202304548

Quantifying Dynamic Phenotypic Heterogeneity in Resistant *Escherichia coli* under Translation-Inhibiting Antibiotics

*Haishuang Zhu, Yixiao Xiong, Zhenlong Jiang, Qiong Liu and Jin Wang**

Supporting Information

Quantifying Dynamic Phenotypic Heterogeneity in Resistant E. coli under Translation-inhibiting Antibiotics

*Haishuang Zhu†, Yixiao Xiong †, Zhenlong Jiang†, Qiong Liu, Jin Wang**

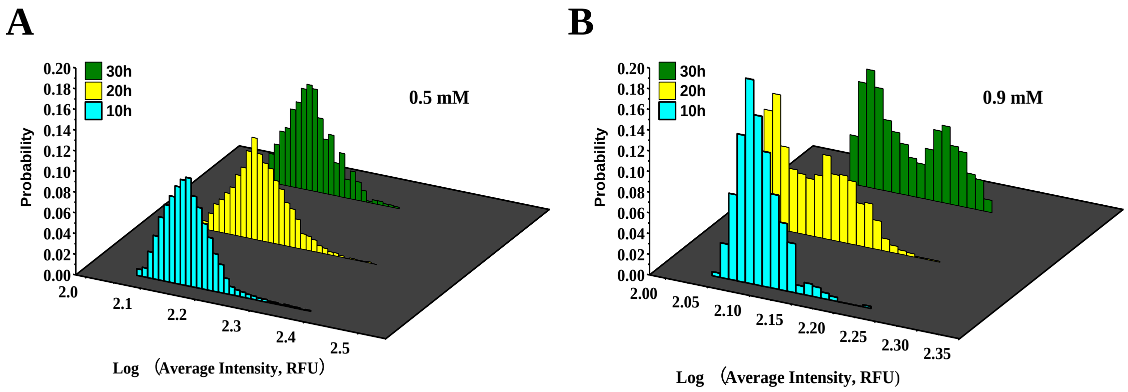

Figure S1. A comparison of the fluorescence intensity distribution between the 0.5 mM Cm and the 0.9 mM Cm experimental groups. The data of 2000 bacteria cells induced by (A) 0.5 mM Cm and (B) 0.9 mM, at 10 h, 20 h and 30 h were shown, respectively.

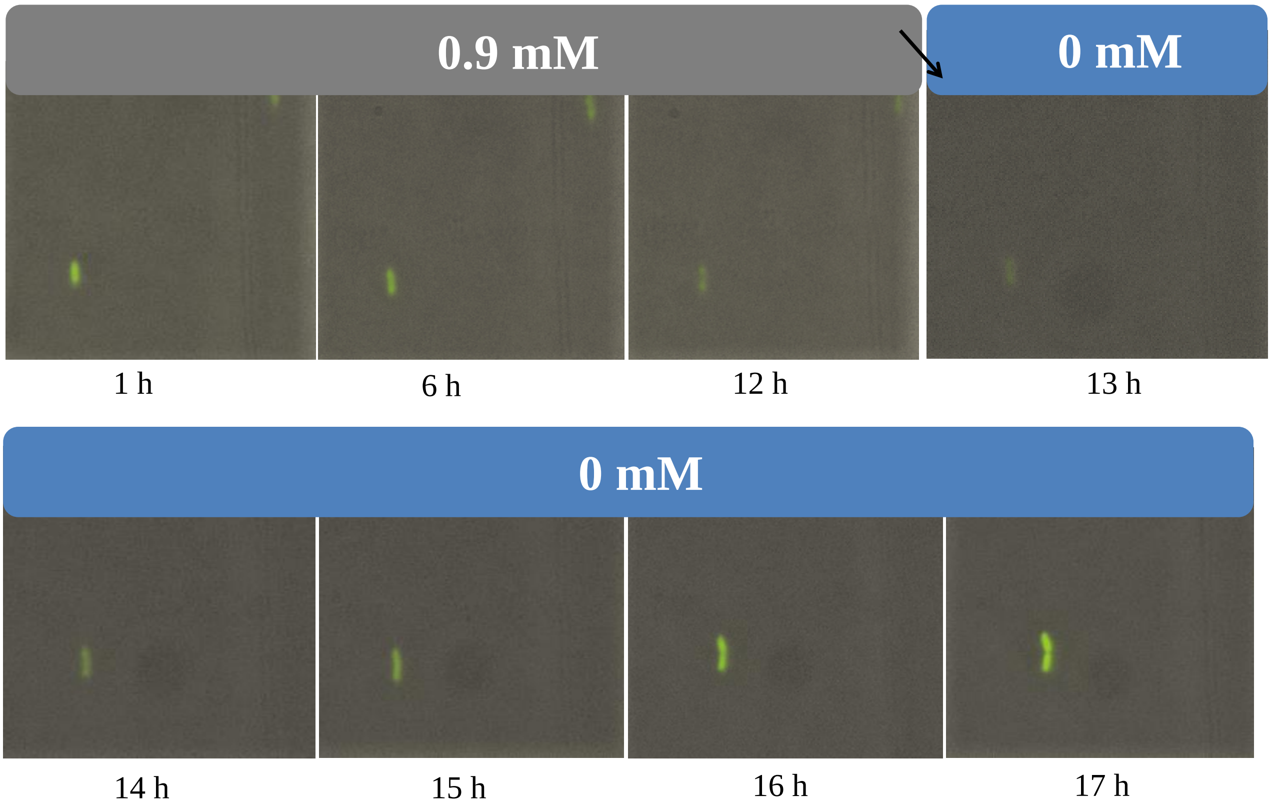

Figure S2. A typical cell recovered to grow and started dividing after treatment of 0.9 mM Cm for 12 h.

Table S1. A comparison of the transition probabilities between the states characterized by either one or two cellular traits at different Cm concentrations. The transition probabilities were calculated over 12-minute time intervals.

		**Transition probability between two states characterized by one cellular trait**					**Total transition probability between four states characterized by two cellular traits**			
		**0.6 mM**	**0.7 mM**	**0.8 mM**	**0.9 mM**		**0.6 mM**	**0.7 mM**	**0.8 mM**	**0.9 mM**
**Fluorescence dimension**	**Dim to Brigh**t	0.0209	0.0692	0.0526	0.0091	**[S/L-CT, L-FL] to [S/L-CT, H-FL]**	0.0482	0.0357	0.0359	0.0212
	**Bright to Dim**	0.0047	0.0080	0.0036	0.0047	**[S/L-CT, H-FL] to [S/L-CT, L-FL]**	0.0019	0.0124	2.9387 e-25	0.0052

**Cycle time dimension**	**Short to Long**	0.0332	0.0161	0.0088	0.0120	**[S-CT, L/H-FL] to [L-CT, L/H-FL]**	0.0303	0.0183	0.0093	0.0217
	**Long to Short**	0.0048	0.0075	0.0048	0.0017	**[L-CT, L/H-FL] to [S-CT, L/H-FL]**	0.0055	0.0142	0.0057	0.0043

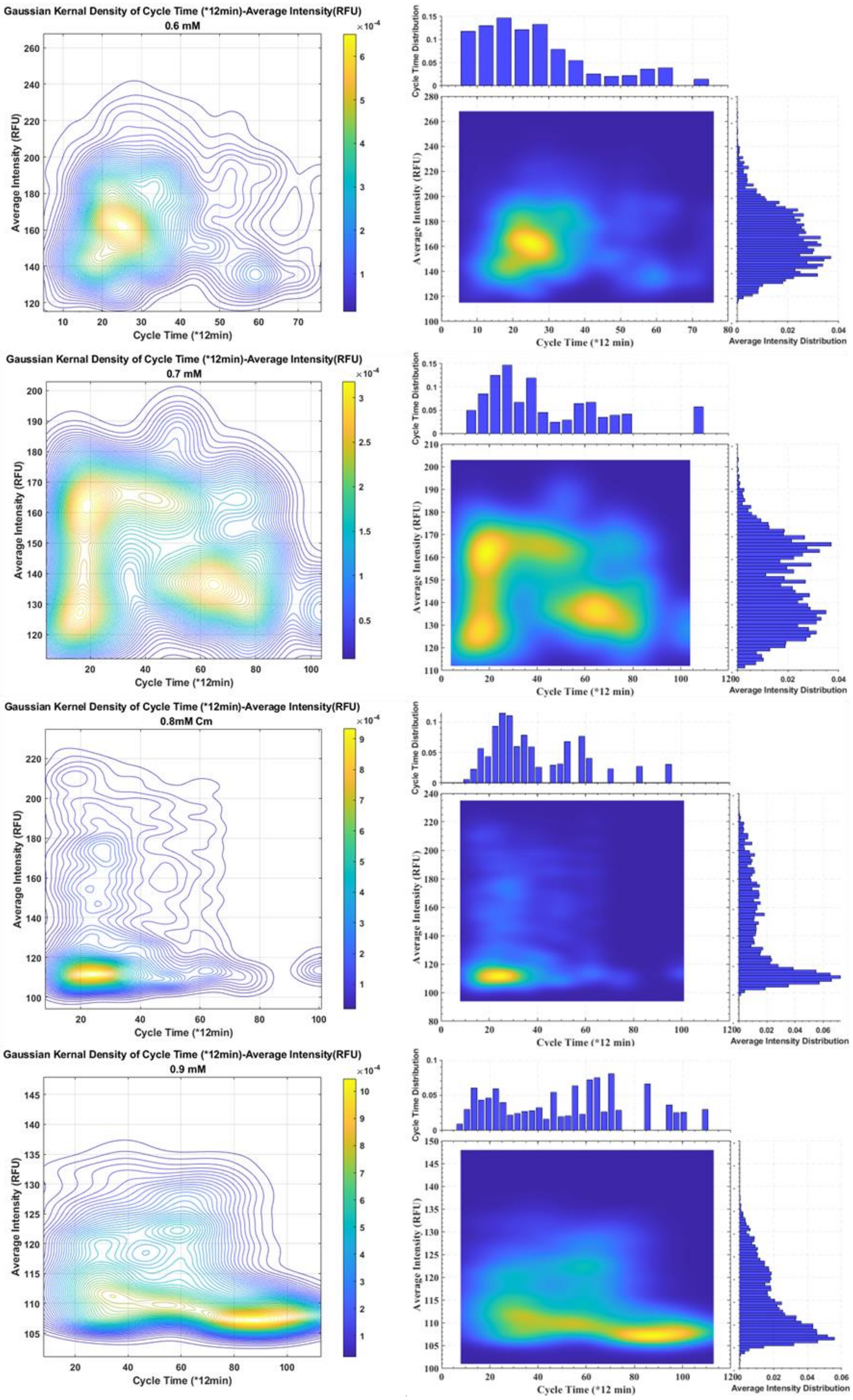

Figure S3. Gaussian Kernel Density Estimation (KDE) contour plots and mesh plots with marginal distributions of the cycle time (*12 min)-average YFP fluorescence intensity (RFU) data under 0.6 mM, 0.7 mM, 0.8 mM, 0.9 mM Cm treatment (from top to bottom).

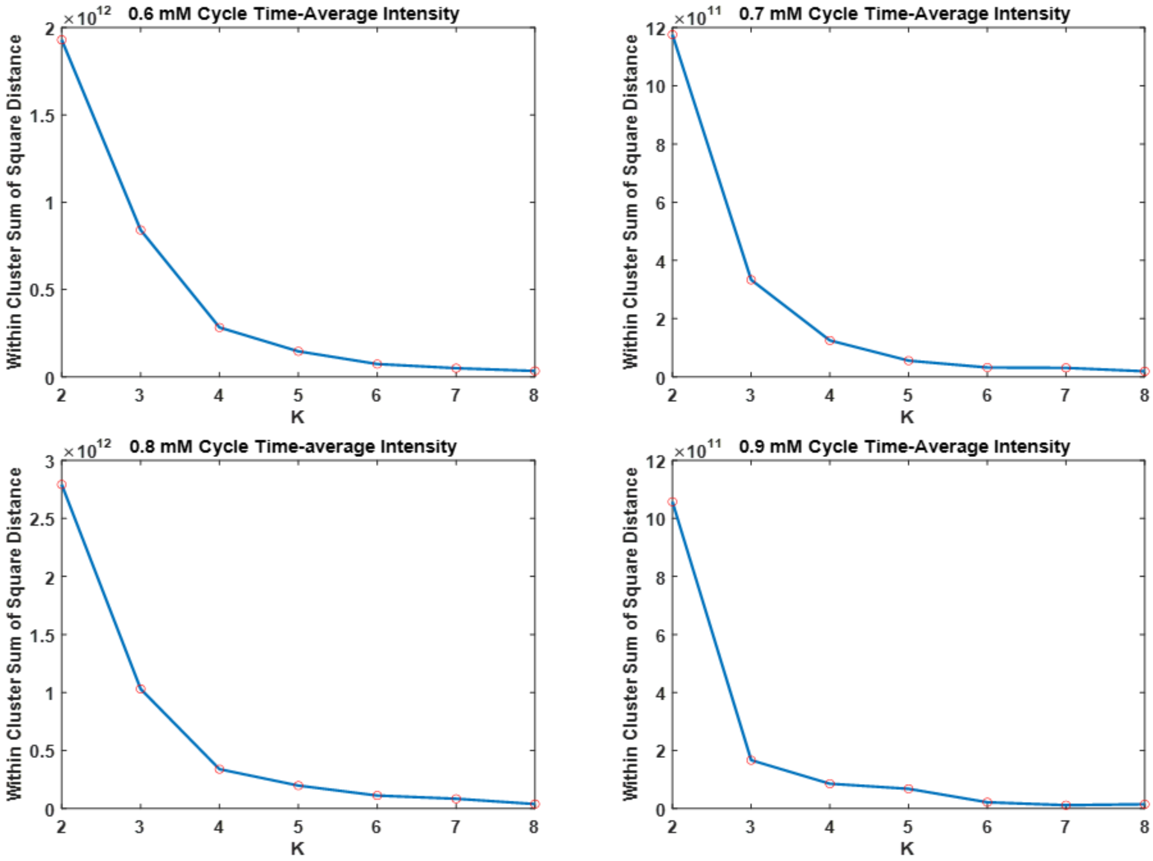

Figure S4. Results from the Elbow method based on K-means clustering of the cycle time (*12 min) - average intensity (RFU) data under 0.6 mM, 0.7 mM, 0.8 mM, 0.9 mM Cm treatment.

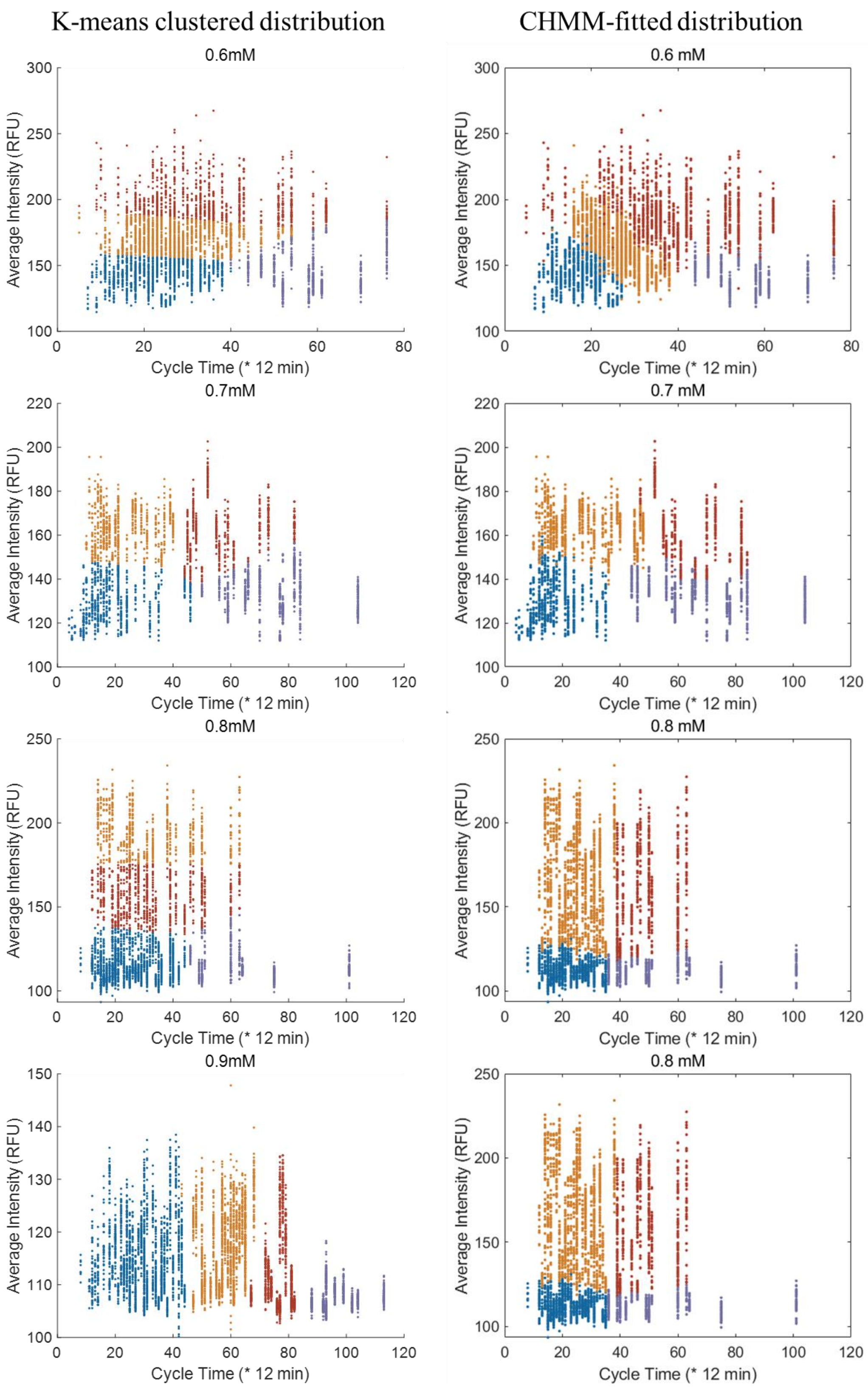

Figure S5. K-means clustered distribution and CHMM-fitted distribution of the cycle time (* 12 min)-average intensity (RFU) data points under diferent concentrations of Cm treatment. Each state is characterized by a color. The K-means clustering was used solely for confirming the appropriate number of states (via the elbow method) and did not influence the subsequent analysis.

Table S2. The switching time (hours) between either two states under 0.6 mM, 0.7 mM, 0.8 mM and 0.9 mM, respectively.

'/' represents the transition is not observed in the HMM trajectories. Numbers 1,2,3,4 refer to [S-CT, L-FL], [S-CT, H-FL], [L-CT, H-FL] and [L-CT, L-FL], respectively.

	0.6 mM			0.7 mM			0.8 mM			0.9 mM		
	1-2	1-3	1-4	1-2	1-3	1-4	1-2	1-3	1-4	1-2	1-3	1-4
Switching Time (hours)	3.9268	/*	/	5.8769	/	/	7.5840	12	12	5.9867	4.1667	4.1667
	2-1	2-3	2-4	2-1	2-3	2-4	2-1	2-3	2-4	2-1	2-3	2-4
Switching Time (hours)	7.6000	4.0359	/	/	6.2000	/	/	2.1200	/	1.3000	4	/
	3-1	3-2	3-4	3-1	3-2	3-4	3-1	3-2	3-4	3-1	3-2	3-4
Switching Time (hours)	/	4	4.8000	4.4667	9.2000	1	/	/	/	2.4000	9.8500	/
	4-1	4-2	4-3	4-1	4-2	4-3	4-1	4-2	4-3	4-1	4-2	4-3
Switching Time (hours)	/	/	4.1500	/	3	6.6833	9.5000	/	4.7000	11.4000	/	5.8571

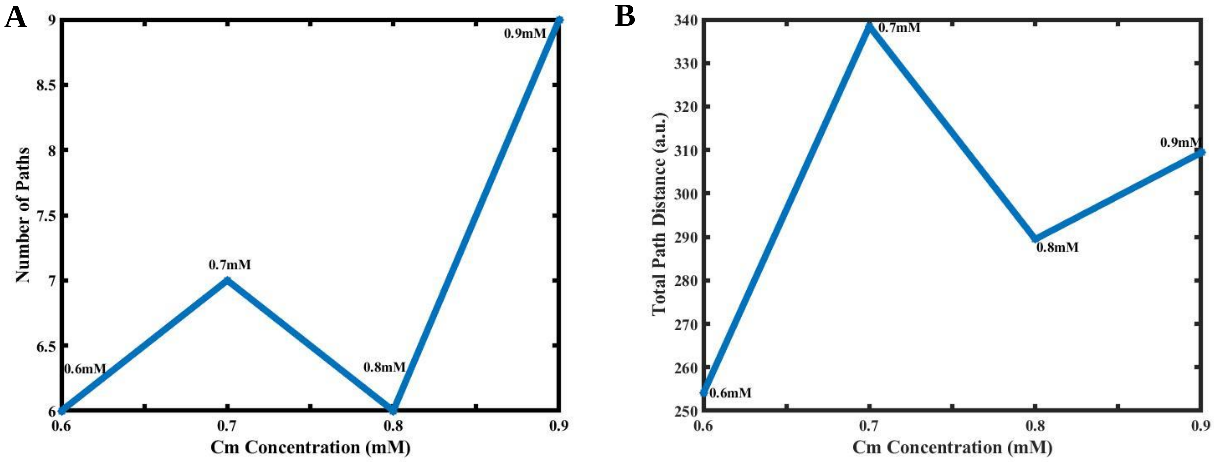

Figure S6. (A) Number of switching paths and (B) total switching path distance vs different Cm concentrations.

Table S3. The mean values, corresponding residence time, transition probabilities, and instantaneous transition rates of the two states after average fluorescence intensity and cell area correlation, obtained from CHMM analysis. The transition probabilities and transition rates were calculated over 12-minute time intervals.

	State mean value (Cell area, average intensity (RFU))		Residence time (%)		Transition probability		Transition Rate (1/minute)	
	State 1	State 2	State 1	State 2	State 1 to State 2	State 2 to State 1	State 1	State 2
**0.6 mM**	116.13, 183.18	142.29, 145.96	48.81 (6.25 h)	51.19 (6.55 h)	0.0046	0.0188	0.0004	0.0016
**0.7 mM**	127.31, 163.18	138.80, 132.15	46.01 (9.36 h)	53.99 (10.98 h)	0.0041	0.0146	0.0003	0.0012

**0.8 mM**	101.45, 166.30	161.85, 112.93	41.66 (6.07 h)	58.34 (8.50 h)	2.7638e-15	0.0151	2.3130e-16	0.0013
**0.9 mM**	94.17, 119.83	190.40, 108.58	45.81 (8.62 h)	54.19 (10.19 h)	0.0012	0.0039	0.0001	0.0003

Table S4. The mean values, corresponding residence time, transition probabilities, and instantaneous transition rates of the two states after cell area and cycle time correlation, obtained from CHMM analysis. The transition probabilities and transition rates were calculated over 12-minute time intervals.

	**State mean value (Cell area, cycle time (×12 min))**		**Residence time (%)**		**Transition probability**		**Transition Rate (1/minute)**	
	State 1	State 2	State 1	State 2	State 1 to State 2	State 2 to State 1	State 1	State 2
**0.6 mM**	117.78, 23.36	150.63, 46.80	65.25 (8.35 h)	34.75 (4.45 h)	0.0109	0.0061	0.0009	0.0005
**0.7 mM**	129.83, 64.13	138.21, 19.19	56.95 (11.58 h)	43.05 (8.76 h)	0.0050	0.0147	0.0004	0.0012
**0.8 mM**	125.86,23.39	144.63, 52.49	62.96 (9.17 h)	37.04 (5.40 h)	0.0058	0.0032	0.0005	0.0003
**0.9 mM**	129.21, 30.77	156.21, 74.33	38.96 (7.33 h)	61.04 (11.48 h)	0.0111	0.0022	0.0009	0.0002

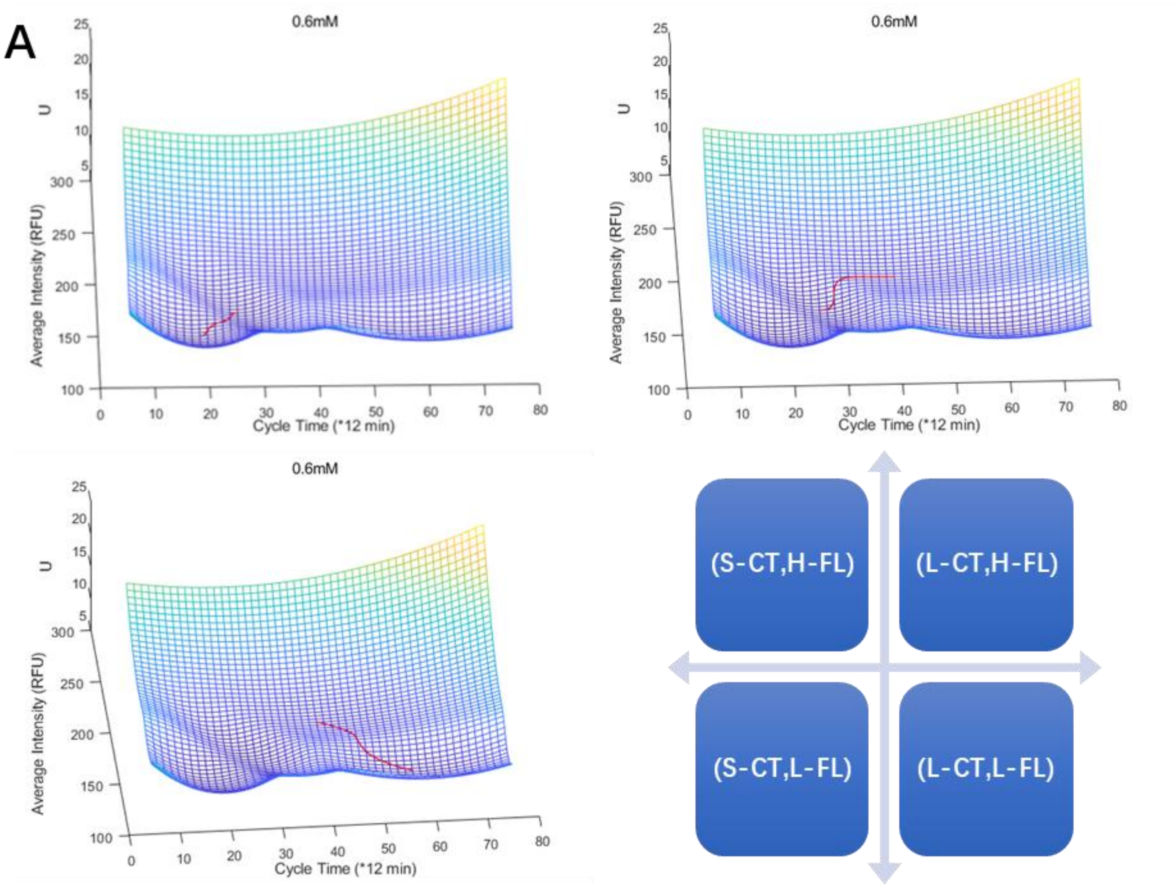

Figure S7. (A) Most probable switching routes (red line) among the four states on the cycle time (×12 min) - average intensity (RFU) landscapes under 0.6 mM Cm inducement.

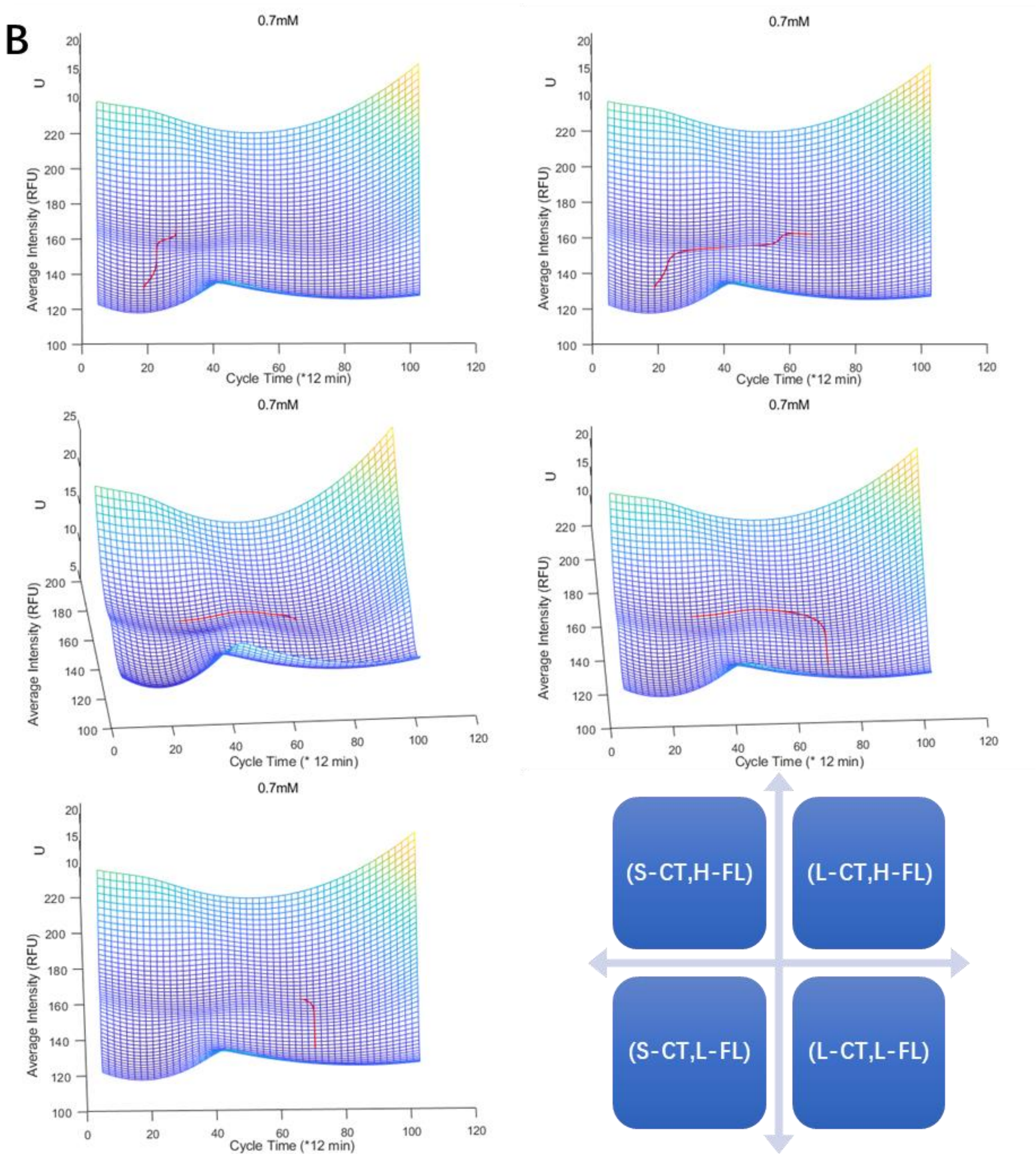

Figure S7. (B) Most probable switching routes (red line) among the four states on the cycle time (×12 min) - average intensity (RFU) landscapes under 0.7 mM Cm inducement.

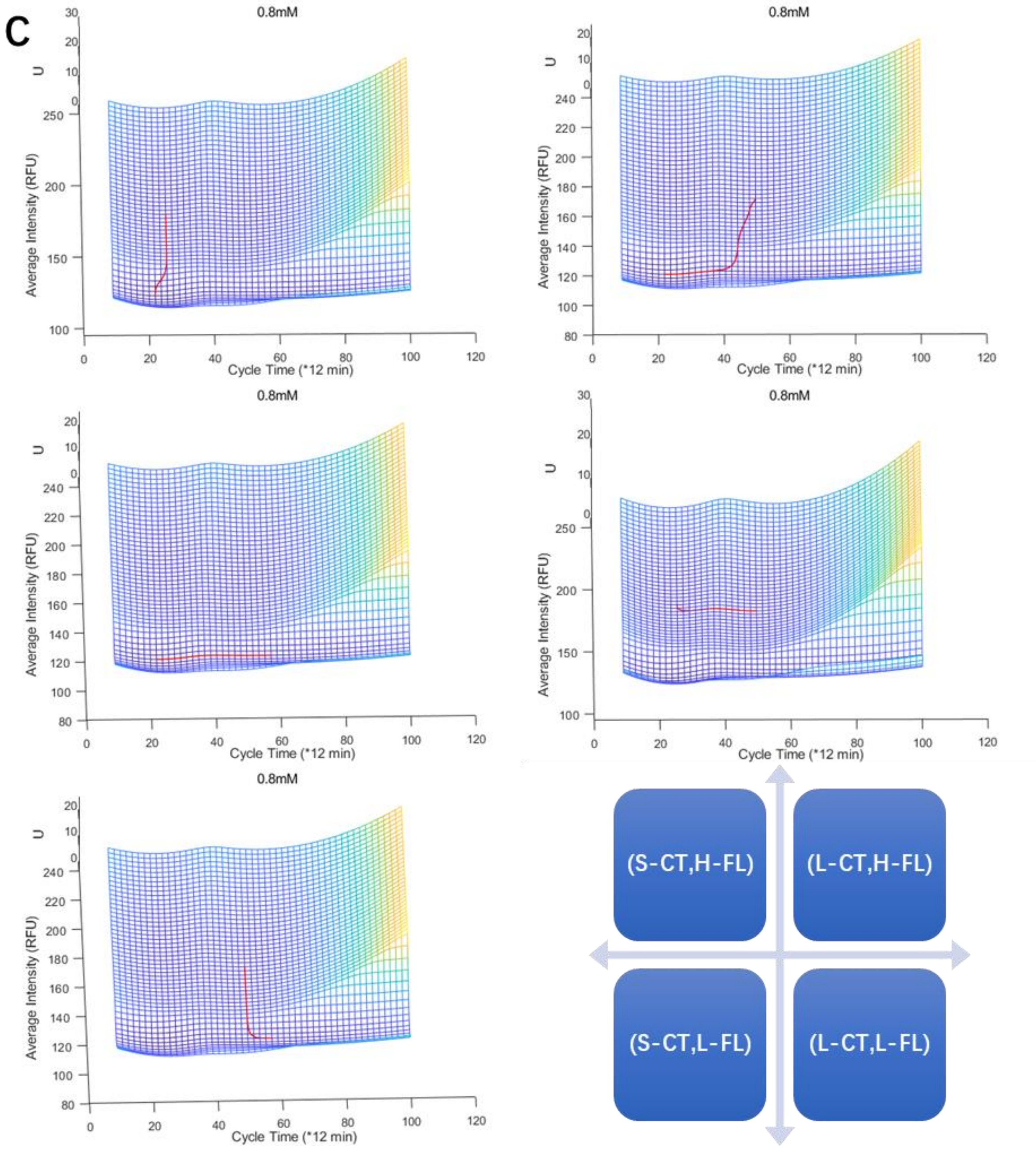

Figure S7. (C) Most probable switching routes (red line) among the four states on the cycle time (×12 min) - average intensity (RFU) landscapes under 0.8 mM Cm inducement.

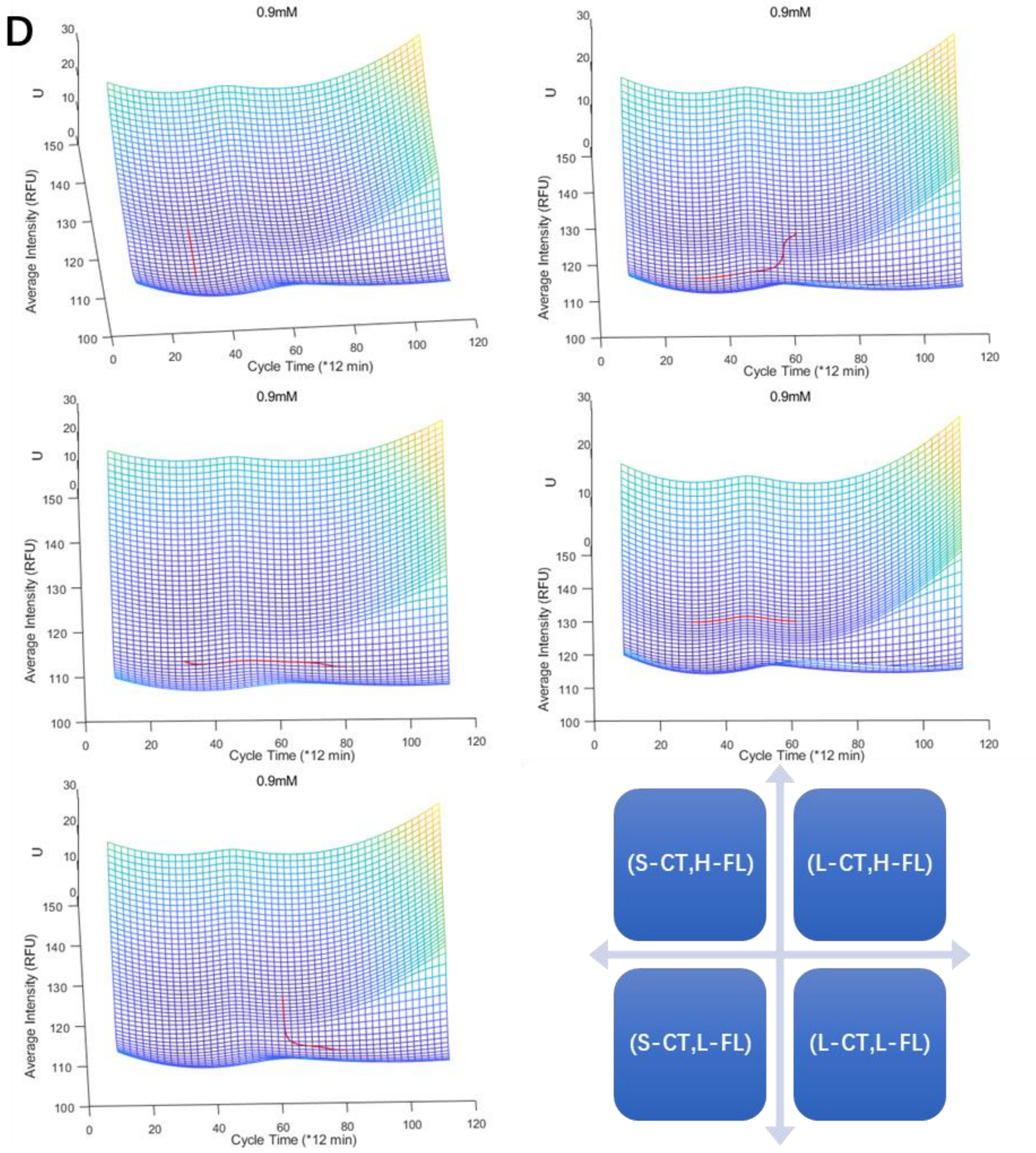

Figure S7. (D) Most probable switching routes (red line) among the four states on the cycle time (×12 min) - average intensity (RFU) landscapes under 0.8 mM Cm inducement.

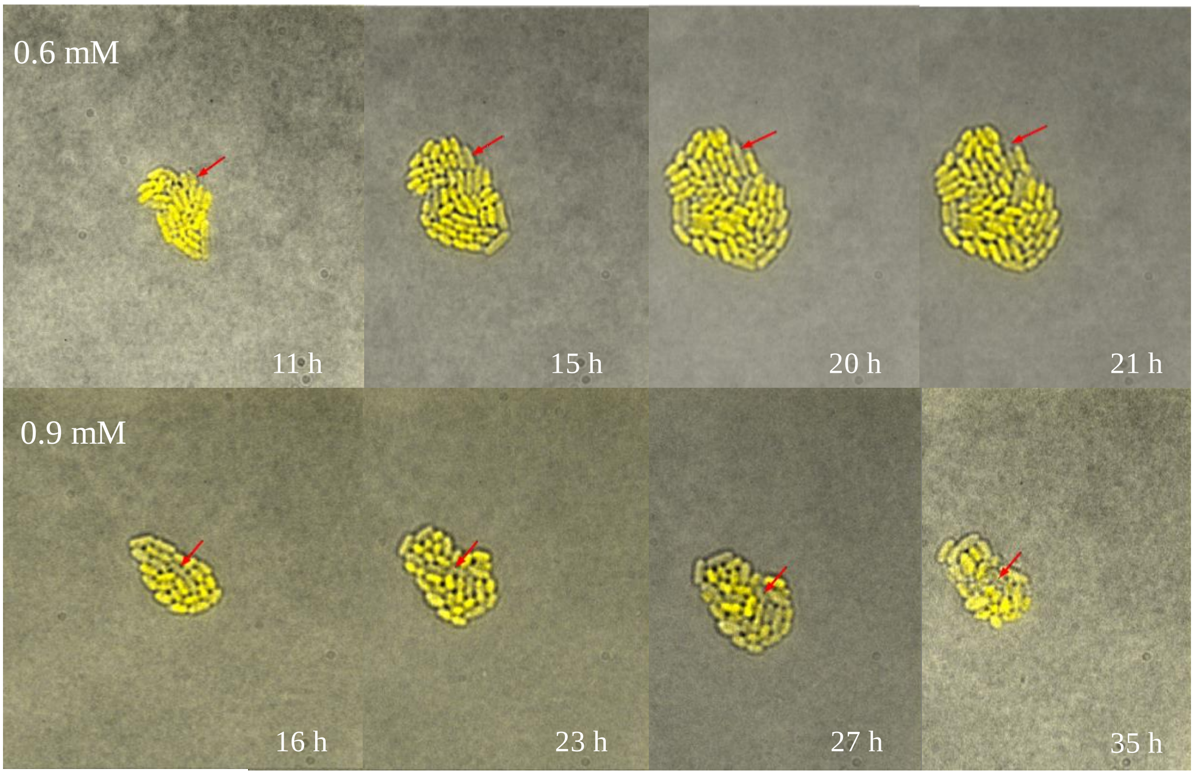

Figure S8. The representative overlay time-lapse images indicated the bacterial cells with dim fluorescence and fast growth rate could be lysed at 0.6 mM Cm and 0.9 mM Cm, respectively.

Movie S1.
Some of the *E. coli* cells in a micro-colony keep dividing in 0.9 mM Cm (bright field).

Movie S2.
Some of the *E. coli* cells in a micro-colony keep dividing in 0.9 mM Cm (fluorescence field).

Movie S3.
The *E. coli* cells keep dormant in 0.9 mM Cm (bright field).

Movie S4.
The *E. coli* cells keep dormant in 0.9 mM Cm (fluorescence field)

Movie S5.
Time-lapse recording of the dynamic behavior of a micro-colony of *E. coli* cells in 0.8 mM Cm (fluorescence field)

Movie S6.
Time-lapse recording of the dynamic behavior of another micro-colony of *E. coli* cells in 0.8 mM Cm (fluorescence field)
